# The Aqueous Extract of *Hemerocallis citrina* Baroni Improves the Lactation-Promoting Effect in Bovine Mammary Epithelial Cells through the PI3K-AKT Signaling Pathway

**DOI:** 10.3390/foods13172813

**Published:** 2024-09-04

**Authors:** Jiaxu Chen, Zhaoping Pan, Qili Li, Yanyang Wu, Xiaopeng Li, Xue Wang, Dandan Hao, Xiaoyu Peng, Lina Pan, Wei Li, Jiaqi Wang, Tao Li, Fuhua Fu

**Affiliations:** 1 Hunan Agricultural Product Processing Institute, Hunan Academy of Agricultural Sciences, Changsha 410125, China; chenjiaxu@hunaas.cn (J.C.); panzhaoping@hunaas.cn (Z.P.); liqili@hunaas.cn (Q.L.); lixiaopeng@hunaas.cn (X.L.); wangxue@hunaas.cn (X.W.); haodandan@hunaas.cn (D.H.); 2 Key Laboratory of Dongting, Yuelushan Center for Industrial Innovation, Changsha 410125, China; 3 College of Food Science and Technology, Hunan Agricultural University, Changsha 410128, China; wuyanyang2002@hunau.edu.cn; 4 Ausnutria Dairy Co., Ltd., Changsha 410200, China; xiaoyu.peng@ausnutria.com (X.P.); lina.pan@ausnutria.com (L.P.); wei.li@ausnutria.com (W.L.); jiaqi.wang@ausnutria.com (J.W.)

**Keywords:** flavonoids, *Hemerocallis citrina* Baroni, hexamethylquercetagetin, lactogenic properties

## Abstract

Insufficient milk supply is a widespread issue faced by women globally and associated with a higher risk of health problems in infants and mothers. *Hemerocallis citrina* Baron, commonly known as daylily, is a perennial edible plant often used in traditional Asian cuisine to promote lactation. However, the active compound(s) and mechanism of its lactation-promoting effect remain unclear. This study aimed to confirm the traditional use of daylily in promoting lactation and investigate its potential active components and underlying molecular mechanisms. Our results showed that the aqueous extracts of *H. citrina* Baroni (HAE) significantly enhanced milk production, and the serum levels of lactation-related hormones, and promoted mammary gland development in lactating rats, as well as increased the levels of milk components in bovine mammary epithelial cells (BMECs) (*p* < 0.05). UHPLC-Q-Exactive Orbitrap-MS analysis revealed that hexamethylquercetin (HQ) is the representative flavonoid component in HAE, accounting for 42.66% of the total flavonoids. An integrated network pharmacology and molecular docking analysis suggested that HQ may be the potential active flavonoid in HAE that promotes lactation, possibly supporting lactation by binding to key target proteins such as STAT5A, PIK3CA, IGF1R, TP53, CCND1, BCL2, INS, AR, and DLD. Cell experiments further demonstrated that HQ could promote cell proliferation and the synthesis of milk proteins, lactose, and milk fat in BMECs. Transcriptomic analysis combined with a quantitative reverse transcription polymerase chain reaction (RT-qPCR) revealed that both HAE and HQ exert a lactation-promoting function mainly through regulating the expression of key genes in the PI3K-Akt signaling pathway.

## 1. Introduction

Insufficient milk supply often leads to an early cessation of exclusive breastfeeding [1]. This shortage is linked to a greater chance of various health problems such as diarrhea, leukemia, and respiratory illnesses in infants and a higher risk of breast and ovarian cancers, diabetes, and heart attacks in mothers [2]. Successful lactation involves a multistage process driven by reproductive and metabolic hormones, including but not limited to estradiol (E_2_), prolactin (PRL), growth hormone (GH), and thyroid hormone [3]. Medications like domperidone and metoclopramide are used to raise PRL levels and potentially increase milk supply [4]. However, given the potential side effects associated with the long-term use of these medicines, including tardive dyskinesia, depression, increased risk of ventricular arrhythmia, and sudden cardiac death [5], finding safe and effective alternatives is of great importance.

*Hemerocallis citrina* Baroni, a perennial plant of the Liliales order, is native to central and northern China, the Korean Peninsula, and Japan. It has been regarded as a functional vegetable crop in traditional Asian cuisine due to its rich nutritional value [6,7]. Recent studies have shown that *H. citrina* is rich in flavonoids, alkaloids, carotenoids, amino acid amides, and other biologically active substances, which are effective in anti-inflammatory, anxiety-relieving, and sleep-promoting effects [8,9,10]. Additionally, *H. citrina* is also known as “Galactogogue” in many Asian countries and is used as an important ingredient in soups to enhance milk production in lactating women [11,12]. Despite its traditional use to promote lactation, there are currently only a few studies that have reported on its lactation-promoting properties. For example, Zhong et al. [12] investigated the effects of *H. citrina* extract on lactation insufficiency in chronic unpredictable mild stress (CUMS) dams and found that the extract could promote weight gain in offspring and increase the levels of PRL and oxytocin in the serum of the model mice. Additionally, network pharmacology predicted that the potential key targets could be MAPK1, STAT3, and TP53. Similarly, Guo et al. [13] studied the therapeutic effects of *H. citrina* extract on a rat model of lactation deficiency induced by bromocriptine. They discovered that the extract significantly increased milk production, as well as the levels of PRL, progesterone, and estradiol, and promoted the repair of mammary gland tissues. Network pharmacology analysis suggested that flavonoid compounds and phenolic substances might be the active ingredients enhancing lactation, primarily acting through the JAK2/STAT5 pathway. However, due to the diversity and complexity of the bioactive substances, no studies have yet systematically validated the potential active components of *H. citrina* for promoting lactation, and its key targets and mechanisms of action remain unclear.

Flavonoids, a subgroup of polyphenols widely present in plants, exhibit multiple physiological and pharmacological activities such as antioxidant, antibacterial, anti-tumor, and anti-cardiovascular disease properties [14,15]. Numerous studies have demonstrated that flavonoids improve lactation performance. For instance, Garavaglia et al. [16] reported an increase in milk production when dairy cows were fed a diet containing silymarin and lycopene for seven consecutive days. Cui et al. [17] found that supplementing the basal diet of dairy cows with 3.0 and 4.5 mg of rutin per kg of diet significantly increased milk production by 10.06% and 3.37%, respectively. Current studies suggest that flavonoid compounds can enhance lactation by binding to the β-estradiol receptor via the α-isoform of membrane-associated estrogen in lactotrophic cells of the anterior pituitary gland and antagonizing dopamine receptors [18]. This leads to up-regulated expressions of PRL receptor (PRLR)-related genes and stimulates mammary gland development, thereby improving lactation performance. However, most existing studies have not elucidated the underlying mechanism of the lactation-promoting effects of *H. citrina* and its flavonoids.

Transcriptomics enables a high-throughput detection of changes in gene expression and provides a comprehensive understanding of the structure and function of genes on a genome-wide scale, thereby revealing the potential mechanisms behind biological effects. Network pharmacology studies functional active substances by constructing a multi-compound, multi-target, and multi-pathway interaction network through the comprehensive analysis of biological system networks. Molecular docking reveals interactions between effective components and potential targets at the molecular level based on the three-dimensional structure of receptor macromolecules and the binding of ligand compounds. The combination of these methods helps to reveal the core targets, metabolites, and pathways in the lactation regulation network.

In this study, first, the lactation-improvement effect of the aqueous extracts of *H. citrina* Baroni (HAE) on lactating rats and bovine mammary epithelial cells (BMECs) was evaluated. Then, UHPLC-Q-Exactive Orbitrap-MS was employed to identify and quantify the active components in HAE responsible for the observed biological effects. Subsequently, transcriptomic analysis was performed to identify genes that are differentially expressed after HAE and hexamethylquercetagetin (HQ) treatment and reveal their corresponding underlying molecular mechanisms involved in lactation processes. Following this, network pharmacology and molecular docking were combined to predict the possible active components in HAE and the core targets related to lactation and model the interactions between HQ and the core target proteins. Finally, a quantitative reverse transcription polymerase chain reaction (RT-qPCR) was employed to clarify the underlying mechanism related to the lactogenic activity.

## 2. Materials and Methods

### 2.1. Chemicals and Reagents

Dried flower buds of *H. citrina* Baroni (Asphodelaceae) were kindly provided by Xinfa Food Co. Ltd. (Qidong, Hunan, China) in July 2022. Methanol and Acetonitrile were LC–MS grade and obtained from CNW Technologies (CNW Technologies GmbH, Teltow, Germany). Ammonium acetate was purchased from Sigma-Aldrich Chemical Co. (St. Louis, MO, USA). HQ was purchased from Yuanye Shengwu Co. Ltd. (Shanghai, China). All other chemicals and reagents employed in this work were of analytical grade.

### 2.2. Preparation of HAE

In total, 100 g *H. citrina* Baroni was ground to powder, extracted twice with 3500 mL of water, and maintained for 20 min in an ultrasonic bath (40 KHz) at 50 °C. The mixture was centrifuged at 10,000 rpm for 15 min at 4 °C and then concentrated by a rotary evaporator (SENCQ R-501, Shenshun Biotechnology Co., Shanghai, China). Finally, the concentrate was lyophilized for 72 h to obtain HAE powder and stored in the refrigerator at −20 °C. LC–MS analysis confirmed that colchicine is not present in the HAE lyophilized powder.

### 2.3. Components in HAE by UHPLC-Q-Exactive Orbitrap-MS Analysis

The separation was performed employing a Vanquish UHPLC system (Thermo Fisher Scientific, Bremen, Germany) in conjunction with an UPLC HSS T3 column (2.1 mm × 100 mm, 1.8 μm). The separated analytes were then detected by an Orbitrap Exploris 120 mass spectrometer (Thermo Fisher Scientific, Bremen, Germany). The mobile phase consists of eluent A (5 mmol/L ammonium acetate and 5 mmol/L acetic acid in water) and eluent B (methanol). The solvent gradient was set as follows: 1% B, 0.7 min; 1–99% B, 9.5 min; 99% B, 11.8 min; 99–1% B, 12 min; 1% B, 15 min. The mass spectrometer was operated in positive/negative polarity mode with a capillary temperature of 320 °C, sheath gas flow rate of 50 arb, auxiliary gas flow rate of 15 arb, and a spray voltage of 3.8 kV (positive) or −3.4 kV (negative).

### 2.4. Experiments Performed with Animals

#### 2.4.1. Experimental Design and Treatments

A total of 12 SD rats (8 ± 2 weeks old) at the beginning of lactation and 120 suckling pups were purchased from Hunan SJA Laboratory Animal Co. Ltd. (license no. SCXK 2023-0004, Changsha, Hunan, China). The animals were placed in standard cages with wood chips and given free access to food and water. The system was set to maintain conditions with a 12 h light and 12 h dark cycle between 22 and 24 °C. Then, 12 lactating rats, each of which had 10 pups, were randomly divided into three groups: the control group (CK), the low-dose aqueous extracts of *H. citrina* Baroni group (HAE-L, 100 mg/kg/day), and the high-dose aqueous extracts of *H. citrina* Baroni group (HAE-H, 300 mg/kg/day). The CK of rats were treated with orally administered distilled water. The experimental protocols were conducted based on the standards of the ARRIVE guidelines, and all animal-care procedures were approved by the Ethical Committee of Hunan Academy of Agricultural Sciences (no. 2023012).

#### 2.4.2. The Weight Gain per Litter and the Organ/Tissue Index of Lactating Rats

Using each litter as the fundamental unit of observation, the pups in each litter were weighed every three days from the 1st day to the 18th day, according to a previous report with some modifications [19]. The weight increase for each litter was determined using the formula: $W_{G}=W_{\left(W\right)}-W_{\left(I\right)}$.

#### 2.4.3. Detection of Serum Hormone Levels

After isoflurane anesthesia, the blood sample was collected with vacuum tubes containing heparin sodium anticoagulant following a cardiac puncture. The blood samples were then centrifuged at 1000 rpm for 15 min at 4 °C, and the serum was stored at −80 °C for subsequent assays. The assays for serum hormones were carried out using the rat PRL, PRLR, and E_2_ enzyme-linked immunosorbent assay (ELISA) kits, respectively (Huangshi Inselisa Biotechnology Co., Ltd., Huangshi, China).

#### 2.4.4. Histopathological Analysis

Histopathological analysis of mammary glands was conducted using an optical microscopy on paraffin material [20]. Briefly, the formalin-treated mammary gland tissues were gradually dehydrated by being immersed in 10% graded ethanol solutions and then embedded in pre-melted paraffin. Sections of 5 µm thickness were produced using a paraffin microtome. The prepared sections were then stained with hematoxylin and eosin (H&E). Finally, mammary tissue morphology of each group was observed and photographed under a microscope Leica digital camera (Leica DFC420C, Leica Mikrosysteme Vertrieb GmbH, Wetzlar, Germany).

### 2.5. Cell Experimentation

#### 2.5.1. Cell Cultures

The immortalized BMECs from the mammary tissues of *Bos taurus* were purchased from WHELAB (Shanghai, China). The cells were subcultured in 90% Dulbecco’s Modified Eagle’s Medium/F12 (DMEM/F12; Gibco, Carlsbad, CA, USA) supplemented with 10% fetal bovine serum (VivaCell, Shanghai, China) and 1% penicillin-streptomycin (VivaCell) in a humidified incubator at 37 °C under an atmosphere with 5% CO_2_. The passage was performed when the cell density reached 90%, and the passage ratio was 1:3.

#### 2.5.2. Cell Proliferation Assay

Cell Counting Kit-8 assay was utilized to assess the effect of HAE and HQ on the viability of BMECs [21]. Briefly, cells were seeded into 96-well microplates (1 × 10^4^ cells/well) to attach at 37 °C and 5% CO_2_ for 24 h. In the CK group, cells were treated with DMEM/F12 medium without HQ. In the treated group, cells were exposed to a medium containing different concentration gradients of HAE (20, 40, 80, and 160 μg/mL) and HQ (100, 200, 400, and 800 μM). After the 48 h incubation times indicated, 10 μL of cell counting kit-8 (CCK-8) reagent (Biosharp, Shanghai, China) was added to the corresponding well and incubated at 37 °C for 2 h. Finally, the absorbance at 450 nm was recorded. The proliferation rate was calculated using the following equation:

$$Proliferation\;rate\left(100\%\right)=\frac{{OD}_{treated}-{OD}_{blank}}{{OD}_{control}-{OD}_{blank}}\times 100$$

#### 2.5.3. Determination of Milk Protein, Lactose, and Milk Fat Content by ELISA

A total of 5 × 10^6^ cells were inoculated into each well of 6-well plates, grown overnight, and treated with different concentrations of HAE (20, 40, 80, and 160 μM) and HQ (100, 200, and 400 μg/mL) for 48 h. Cell debris was eliminated from the culture supernatants by centrifuging them at 3000× *g* for 10 min at 4 °C to remove cell debris, after which they were collected for ELISA to determine the milk protein (α-casein, β-casein, and whey protein), lactose, and milk fat levels according to the manufacturer’s instructions (Fujian Quanzhou Ruixin Biotechnology Co., Ltd., Quanzhou, China). The absorbance values of samples were measured at 450 nm and used to calculate the concentrations with standard curves.

### 2.6. Network Pharmacology Analysis

#### 2.6.1. Screening of HAE Flavonoid Targets and Lactation-Related Targets

The SwissADME platform (http://www.swissadme.ch/, accessed on 28 March 2023) was utilized to screen the HAE flavonoid components detected using UHPLC-Q-Exactive Orbitrap-MS analysis. The screening criteria were as follows: High Gl absorption and positive results in at least 2 of 5 drug property predictions (Lipinski, Ghose, Veber, Egan, Muegge). The targets for potential flavonoid components were screened using the PharmMapper database (http://lilab-ecust.cn/pharmmapper/index.html, accessed on 28 March 2023). Lactation-related targets were obtained through GeneCards and OMIM (http://www.omim.org/, accessed on 28 March 2023), using “Lactation” as the keyword. All targets were standardized as Gene Symbols using the UniProt protein database (https://www.uniprot.org, accessed on 29 March 2023). The intersection of potential targets for HAE flavonoids and lactation-related targets was obtained through Venny 2.1.0 (https://bioinfogp.cnb.csic.es/tools/venny/, accessed on 29 March 2023).

#### 2.6.2. Protein–Protein Interaction (PPI) Network Construction

The PPI network was constructed by submitting the target proteins to the STRING12.0 database (https://string-db.org, accessed on 1 April 2022), with the biological species set to “Homo sapiens”, and the minimal connection score threshold set to 0.700. The network relationship file was imported into Cytoscape 3.7.1 to visualize the PPI network. Potential functional protein modules were then identified using the MCODE plugin for further PPI network analysis.

#### 2.6.3. Core Target Functional Enrichment Analysis

The intersection of potential targets for HAE flavonoids and lactation-related targets, as well as the potential core targets, was uploaded to the Metascape platform (http://metascape.org/gp/index.html, accessed on 1 April 2022) for Gene Ontology (GO) function and Kyoto Encyclopedia of Genes and Genomes (KEGG) pathway enrichment analysis. The results were visualized using an online bioinformatics tool (http://www.bioinformatics.com.cn, accessed on 1 April 2022).

### 2.7. Molecular Docking

The key flavonoid components and potential core targets of HAE were obtained through ITP network analysis. The 3D protein structures of these targets were obtained from the PDB database (https://www.rcsb.org) (accessed on 5 April 2022). Then, these protein structures were imported into PyMOL 3.0.2 software, the targets were dehydrated and hydrogenated, and the irrelevant ligands were removed. The key flavonoid components and receptor proteins were docked using the AutoDockTools program, and the docking results were then imported into PyMOL 3.0.2 software for visualization analysis.

### 2.8. Transcriptomic Analysis

After the BEMCs were treated with a medium containing 400 μg/mL HAE and 80 μM HQ for 48 h, the cells were washed twice with PBS. Then, total RNA was extracted using Trizol reagent (YEASEN Biotechnology Co., Ltd., Shanghai, China) based on the manufacturer’s instructions. The RNA amount and purity were quantified utilizing a NanoDrop ND-1000 spectrophotometer (Thermo Fisher Scientific, Waltham, MA, USA), and the RNA integrity was assessed by 1% agarose gel electrophoresis and a universal hood II transilluminator gel documentation system (BIO-RAD, Hercules, CA, USA). Library preparation and sequencing were performed at Gene Denovo Biotechnology Co. (Guangzhou, China). GO function and KEGG pathway enrichment analysis were performed to analyze the main functions and identify significantly enriched pathways of differentially expressed genes (DEGs), respectively.

### 2.9. DEG Validation by RT-qPCR

The total RNA concentration of 400 μg/mL HAE and 80 μM of HQ-treated cells and CK cells were extracted. The Hifair^®^ III 1st Strand cDNA Synthesis Kit (YEASEN, Shanghai, China) was utilized to synthesize cDNA according to the manufacturer’s instructions. RT-qPCR was performed using a CFX96 Touch real-time PCR detection system (Bio-Rad Laboratories, Hercules, CA, USA) with 10 μL of PCR reaction mixture, including 1 μL of cDNA, 5 μL of Hieff^®^ qPCR SYBR Green Master Mix with Low Rox Plus (Yeasan, Shanghai, China), 0.2 μL of forward primer, 0.2 μL of reverse primer, and 3.6 μL of double-distilled water. The *GAPDH* gene was selected as the reference gene, and the primer sequences are presented in Table S1. The reactions were incubated at 95 °C for 5 min, followed by 40 cycles of 95 °C for 10 s and 60 °C for 30 s. The RT-qPCR data were calculated using the 2^−△△Ct^ method.

### 2.10. Statistical Analysis

Data are expressed as mean ± standard deviation. Statistical analysis and plotting were conducted utilizing GraphPad Prism 8.0 (GraphPad Software, La Jolla, CA, USA). The treatment groups were analyzed using one-way ANOVA followed by Tukey’s post hoc test, and *p* < 0.05 was considered statistically significant.

## 3. Results

### 3.1. HAE Up-Regulated Lactation Performance in Lactating Rats

**Figure 1:**
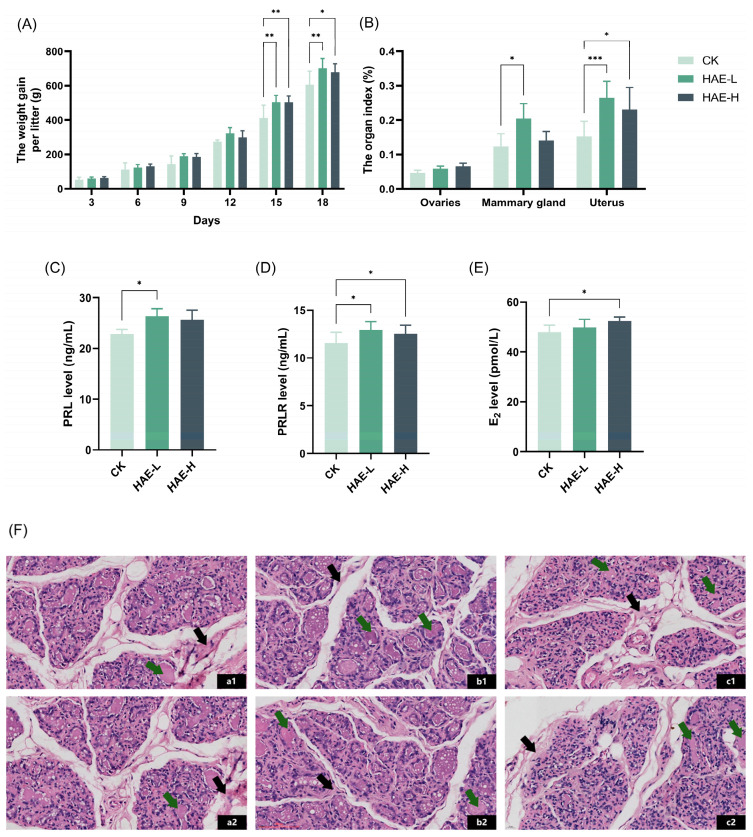

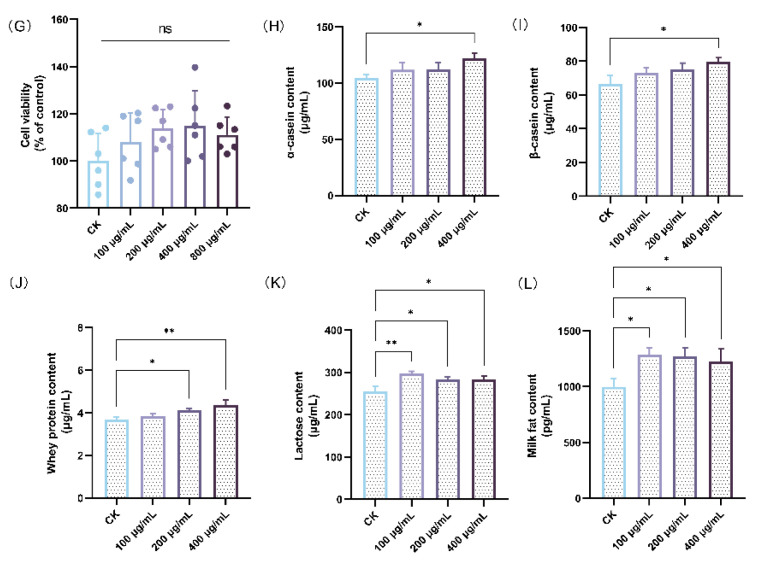
Effect of HAE on lactation function. (**A**) The weight gain per litter of pups during the lactation. (**B**) The ovaries, mammary glands, and uterus index of lactating rats. (**C**) The serum prolactin (PRL) level of lactating rats. (**D**) The serum prolactin receptor (PRLR) level of lactating rats. (**E**) The serum estradiol (E_2_) level of lactating rats. (**F**) Mammary gland structure (40 magnification, H&E stained section) of lactating rats from the CK group (a1,a2), the HAE-L group (b1,b2), and the HAE-H group (c1,c2). The green arrows indicate pink secretions; the black arrows indicate fibrous connective and adipose tissues. (**G**) The effect of various concentrations of HAE on the cell viability of BMECs. The contents of α-casein (**H**), β-casein (**I**), whey protein (**J**), lactose (**K**), and milk fat (**L**) of samples from the HAE group and the CK group. ns represents not significant. * *p* < 0.05, ** *p* < 0.01, *** *p* < 0.001.

To explore the lactation-promoting activity of HAE in lactating rats, the litter weight of the pups was measured, and the weight gain was calculated as a response variable that indirectly reflects the amount of lactation. As shown in Figure 1A, there was no significant difference in the average litter weight gain of the pups in comparison to the CK group on days 3–12 of the lactation period. However, pups in the two groups receiving HAE supplementation experienced a higher average weight gain per litter compared to the CK group on days 15–18 (*p* < 0.05), among which the HAE-L group presented a better effect. The litter weight of the pups in the HAE-L group was enhanced by 15.8% in comparison to the CK on the last day of lactation. The results for the organ/tissue index of the lactating rats are shown in Figure 1B. The mammary gland tissue index of the lactating rats in the HAE-L group was significantly increased compared to the CK group (*p* < 0.05), which was enhanced by 1.2 times. The uterus index increased significantly in both the HAE-L and HAE-H groups (*p* < 0.05), while the enhancement was more obvious in the HAE-L group. There was no significant difference in the ovarian index between groups. These results suggest that HAE, especially at low doses, can promote weight gain in pups and contribute to lactation. ELISA was used to assess the effects of HAE on lactation-related hormone levels in the serum of lactating rats. As shown in Figure 1C–E, HAE increased the serum hormone levels of PRL, PRLR, and E_2_ in comparison to the CK group. Specifically, the HAE-L group showed significantly higher levels of PRL and PRLR compared to the CK group (*p* < 0.05), with increases of 15.15% and 11.95%, respectively. In the HAE-H group, the levels of PRLR and E_2_ were significantly higher than the CK group (*p* < 0.05), increasing by 8.65% and 9.27%, respectively. Histopathological analysis was utilized to investigate the impact of HAE on the mammary gland structure of lactating rats. As shown in Figure 1F, mammary gland sections from the various treatment groups displayed a typical lactation-stage appearance. Specifically, in the CK group, the acinar and ductal lumens were significantly expanded, and most of the acinar lumens contained pink secretions. Additionally, fibrous connective tissue and adipose tissue were interspersed throughout the lobules. In contrast, the HAE-L group showed well-developed mammary tissue characterized by enlarged and densely distributed acinar cavities and clearly defined lobules. Many acini were filled with secretions, and the amount of fibrous connective tissue and fatty tissue was significantly reduced, suggesting active milk secretion within the mammary tissue. Meanwhile, in the HAE-H group, the mammary gland tissue showed extended ducts and irregular-shaped acini. The lobular structure was less distinct, with fibrous connective and fatty tissue spread within the stroma of the mammary gland.

### 3.2. HAE Increased Milk Protein, Lactose, and Milk Fat Contents in BMECs

The effects of HAE on lactation were investigated using a BMEC model to determine its capability to stimulate milk production. Initially, the CCK-8 assay was used to detect the effect of different concentrations of HAE (100–800 μg/mL) on the cell viability. As shown in Figure 1G, all concentrations of HAE promoted the proliferation of BMECs in comparison to the CK group. However, at a concentration of 800 μg/mL, cell viability was reduced to a certain extent compared to 400 μg/mL. Therefore, the selected range for HAE was determined to be 0–400 μg/mL. The effects of varying concentrations of HAE (0–400 μg/mL) on milk protein (α-casein, β-casein, and whey protein), lactose, and milk fat content in BMECs was assessed by ELISA. As shown in Figure 1H,I, the casein content was significantly increased after 400 μg/mL HAE treatment (*p* < 0.05). The contents of α-casein and β-casein were 122.29 and 79.56 μg/mL, respectively, which were 1.17 and 1.20 times that of the CK group. Figure 1J showed that all of the concentrations of HAE enhanced whey protein content in a dose-dependent manner, with significant (*p* < 0.05) and highly significant (*p* < 0.01) enhancements at 200 and 400 μg/mL, respectively. Figure 1K,L revealed that treatment with different HAE concentrations significantly increased lactose and milk fat content (*p* < 0.05), with the highest values of 297.05 μg/mL and 1282.19 pg/mL, respectively, observed at 100 μg/mL.

### 3.3. RNA-seq Revealed the Regulatory Mechanism of HAE on the Lactation-Promoting Effect of BMECs

**Figure 2:**
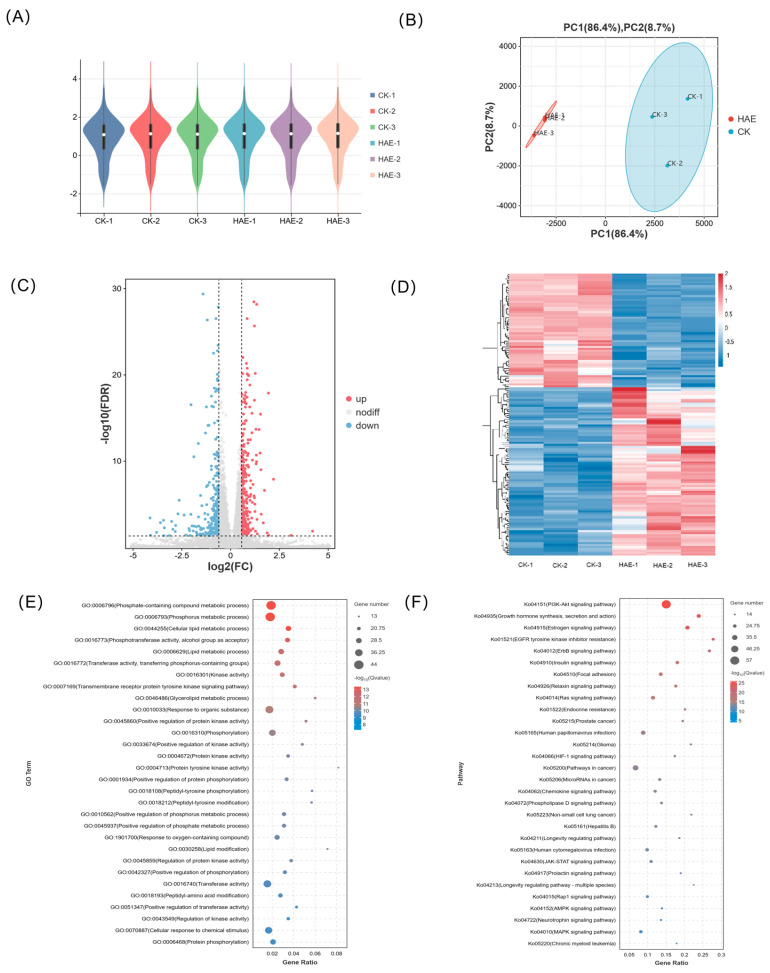
Transcriptome analysis of BMECs from the HAE group and the CK group. (**A**) Violin plot for gene expression. The *x*-axis of the plot represents different samples; the *y*-axis represents the logarithmic scale of FPKM expression levels for the samples. (**B**) Principal component analysis (PCA) diagram. The percentages indicate the contribution of each principal component to the variance in the dataset. (**C**) Volcano plot of DEGs. Genes expressed at higher levels are shown in red; genes expressed at lower levels are shown in blue. (**D**) Clustering heat map of DEGs. Hierarchical clustering is based on log_10_ (FPKM + 1). (**E**) GO enrichment analysis of DEGs. (**F**) KEGG enrichment analysis of DEGs.

The effect of HAE (400 μg/mL) on the transcript profile in BMECs was conducted to investigate the potential mechanisms behind the lactogenic performance. Following the filtration of raw data, examination of sequencing error rates, and analysis of guanine–cytosine content, a total of 43.63 Gb of clean data were generated, averaging 7.27 Gb for each sample. The Q30 base percentage ranged from 90.86 to 93.00%, and the GC content varied from 51.56 to 52.26%, indicating an evenly distributed base and high sequencing accuracy (Table S2). The alignment efficiency is the most direct reflection of transcriptome data utilization. The rate of the successful alignment of sequenced reads to the genome exceeded 96.14%, with a matching efficiency exceeding 93.68%, showing the high accuracy of the sequencing (Table S3). The FPKM distribution violin diagram of the samples showed a low variability in gene expression levels within each sample and high overall expression levels (Figure 2A). The PCA results showed that the samples between the HAE group and the CK group were well separated, representing significant differences between samples from different treatments (Figure 2B). The combined contribution rate of PC1 and PC2 was 95.1%, reflecting strong parallelism within the groups. Based on the differential analysis criteria of FoldChange > 1.2 and FDR < 0.05, a total of 2489 genes were identified as DEGs, with 1520 significantly up-regulated and 969 significantly down-regulated. In the volcano plot, different expressions of genes were represented using different colors (Figure 2C). The clustering heat map of each group differentiated is shown in Figure 2D. Combined with the PCA, significant differences in the expression patterns of genes were observed in the BMECs between the HAE group and the CK group. The enrichment analysis of all DEGs was conducted through GO and KEGG enrichment analyses; the results are shown in Figure S1.

The lactation process involves complex molecular systems and pathways, including the secretion of lactation-related hormones and the proliferation and specialization of acinar epithelial cells, along with the synthesis and secretion of milk constituents. To understand how HAE influences the molecular pathways related to lactation, a total of 125 DEGs associated with the lactation process in the CK group vs. the HAE group is presented in Table S4, and the GO and KEGG enrichment analyses of these DEGs were conducted. The GO enrichment analysis of DEGs related to lactation process is shown in Figure 2E. These DEGs were mainly enriched in the phosphate-containing compound metabolic process (GO:0006796), phosphorus metabolic process (GO:0006793), cellular lipid metabolic process (GO:0044255), phosphotransferase activity, alcohol group as acceptors (GO:0016773), and lipid metabolic process (GO:0006629). These pathways are crucial for the metabolic activities and cellular processes necessary for lactation, emphasizing the role of phosphorus and lipid metabolism in milk production and secretion. The KEGG enrichment analysis of DEGs related to lactation process is shown in Figure 2F. These DEGs were mainly enriched in the PI3K-Akt signaling pathway (ko04151), growth hormone synthesis, secretion and action (ko04935), estrogen signaling pathway (ko04915), EGFR tyrosine kinase inhibitor resistance (ko01521), and ErbB signaling pathway (ko04012). Among the top 30 enriched pathways, the PI3K-Akt signaling pathway was found to be the most significant pathway with the highest number of genes and is known for its role in cell growth and survival, indicating its potential role in the lactogenic effects of HAE.

### 3.4. Identification of the Compounds in HAE Based on UHPLC-Q-Exactive Orbitrap-MS

**Figure 3:**
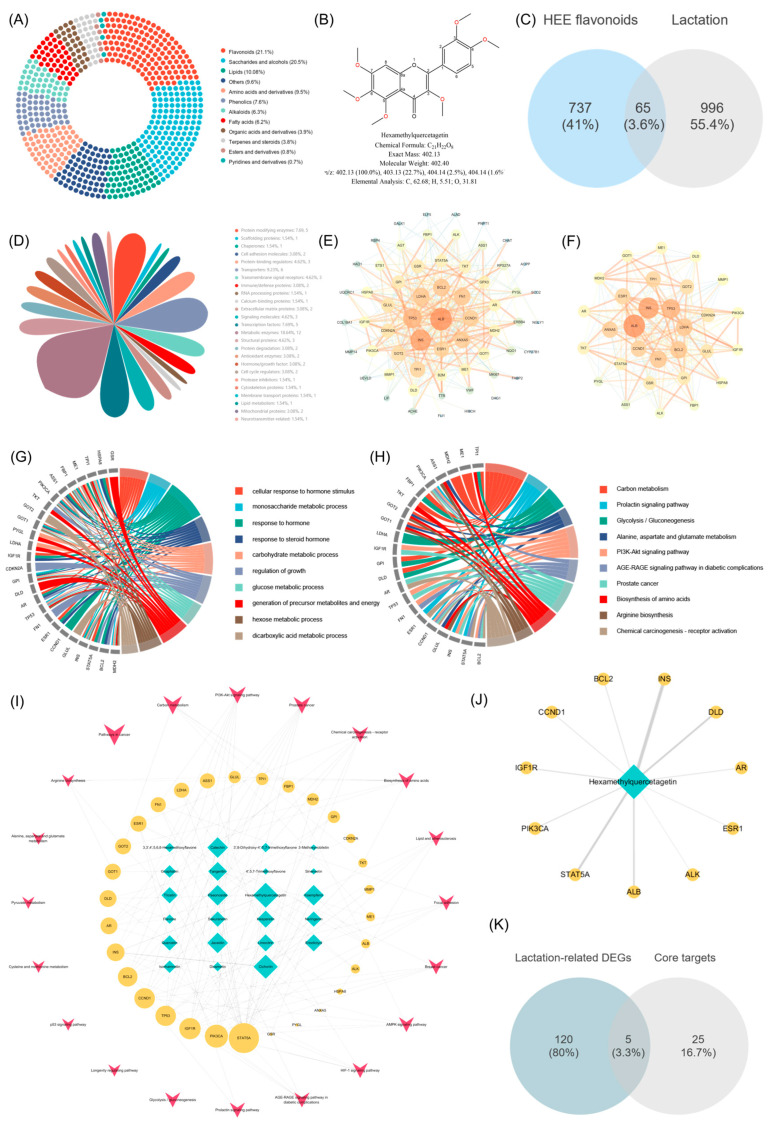
Results of UHPLC-Q-Exactive Orbitrap-MS analysis and network pharmacology analysis of HAE. (**A**) The proportional representation of various compound categories in HAE. (**B**) The structure of HQ. (**C**) The Venn diagram of the intersection between HAE flavonoids and lactation. (**D**) Target classification. (**E**) PPI network. Targets with larger nodes in the network have higher degree values; the thickness of edges represents interactions between targets. (**F**) PPI network clusters. GO (**G**) and KRGG (**H**) enrichment analysis of the potential functional protein modules after cluster analysis using the MCODE plugin. (**I**) ITP network. The blue–green nodes are potential flavonoid components; the yellow nodes are the potential functional protein modules; and the red dots are the KEGG signaling pathway. Larger node sizes indicate higher degree values. (**J**) The main targets of HQ. The thickness of edges represents interactions between targets. (**K**) The Venn diagram of the intersection between 125 lactation-related DEGs based on RNA-Seq and 30 potential core targets.

UHPLC-Q-Exactive Orbitrap-MS was employed for a comprehensive characterization of the compounds in HAE. The total ion current (TIC) diagrams of the compounds analyzed using both ESI+ and ESI- modes are shown in Figure S2. As a result, 499 compounds were identified, including mainly alkaloids (22), amino acids and derivatives (58), esters and derivatives (21), fatty acids (43), flavonoids (75), lipids (22), organic acids and derivatives (50), phenolics (78), pyridines and derivatives (8), saccharides and alcohols (43), terpenes and steroids (21), and others (58). The donut chart illustrated the proportional representation of various compound categories within the total composition (Figure 3A). The results showed that flavonoids had the highest relative content and occupied the largest proportion of the analyzed compounds at 21.1%. The peak areas of all flavonoids were further compared, and the top 20 flavonoids are shown in Table 1. Among them, HQ was identified as the flavonoid with the highest relative content, significantly exceeding other flavonoids and accounting for 42.66% of the total flavonoid content. HQ is a polyethoxylated flavonoid, with its structure shown in Figure 3B. It has six methoxy groups (-OCH3) at positions 5, 6, 7, and 8 on the benzopyran ring and positions 2′ and 4′ on the phenyl ring, which may help define the chemical properties and biological activities of HAE.

### 3.5. Network Pharmacology Combined with RNA-seq Revealed HAE Flavonoid-Promoting Effects through Potential Core Targets

The SwissADME platform was used to screen the HAE flavonoid components detected in the above section, resulting in a total of 23 potentially active flavonoids. The targets for potential flavonoid components are screened using the PharmMapper platform, yielding 737 targets. Lactation-related targets were obtained through GeneCards and OMIM, and 996 lactation targets were generated after removing duplicates. The 737 targets of HAE flavonoids intersected with the 996 lactation targets, resulting in 65 potential therapeutic targets (Figure 3C). The details of the 65 targets are listed in Table S5. These targets were further classified, with the highest proportion being metabolic enzymes (18.64%) (Figure 3D). These results indicate that the metabolic enzymes play crucial roles in various biochemical pathways involved in lactation, and the potential modulation of these enzymes by HAE flavonoids may contribute to their therapeutic effects. Furthermore, these 65 intersection targets were uploaded to the STRING12.0 database to obtain the PPI network information. After hiding the targets without relevant connection, a network containing 61 nodes and 362 edges was constructed (Figure S3), which was subsequently reconstructed using the Cytoscape 3.7 software (Figure 3E). Using the MCODE plugin to further analyze the PPI network, a potential functional protein module consisting of 30 notes and 206 edges was obtained (Figure 3F). Targets with larger nodes in the network have higher degree values.

The GO and KEGG enrichment analyses of the intersection targets (Figure S4A,B) and core targets (Figure 3G,H) were conducted using the Metascape platform. Detailed information on the top 30 KEGG signaling pathways of the intersection of potential targets for HAE flavonoids and lactation-related targets, as well as the potential core targets, is in Tables S6 and S7, respectively. The GO enrichment analysis of the potential core targets is shown in Figure 3G. These targets were mainly enriched in response to hormone (GO:0009725), carbohydrate metabolic process (GO:0005975), and glucose metabolic process (GO:0006006). The results suggested that the potential core targets identified in this study may play critical roles in coordinating hormonal signaling and metabolic processes for successful lactation. The KEGG enrichment analysis of the potential core targets is shown in Figure 3H. The results showed that, among the top ten KEGG pathways, pathways closely related to the lactation process include amino acid biosynthesis (hsa04510), the prolactin signaling pathway (hsa00630), and the PI3K-Akt signaling pathway (hsa01210). The enrichment of the amino acid biosynthesis pathway revealed the importance of these core targets in regulating the synthesis of amino acids, which are the building blocks of milk proteins. The enrichment of the prolactin signaling pathway showed that these core targets could have direct implications for the initiation and sustenance of lactation. Additionally, the PI3K-Akt signaling pathway is a well-known regulator of cell growth, proliferation, and survival, all of which are important for mammary gland development and lactation. This pathway was also identified as the top enriched pathway in the previous transcriptome KEGG enrichment analysis. This consistent finding further highlights the central importance of the PI3K-Akt pathway in modulating the effects of HAE flavonoids on lactation-related processes.

Based on the KEGG analysis results of potential core targets, an ingredient-target-pathway network (ITP) consisting of 30 core targets, 23 flavonoid components, and 20 pathways was constructed (Figure 3I). Built-in tools in Cytoscape 3.7 software were employed to analyze the topological parameters of the network, revealing the core ingredients and core targets. The results showed that HQ, cichoriin, paeonoside, jaceidin, catechin, kaempferol, and tangeritin were predicted as the potential core flavonoids (Table S8). Among them, HQ had the highest degree value and closeness centrality, indicating that HQ may be the core flavonoid component of HAE in promoting lactation. The network of HQ is displayed in Figure 3J. In addition, STAT5A, PIK3CA, IGF1R, TP53, CCND1, BCL2, INS, AR, and DLD were predicted as the potential core targets (Table S9). The 30 potential core targets were further intersected with the 125 lactation-related DEGs previously obtained through RNA-seq. Five gene symbols including BCL2, ESR1, FN1, PIK3CA, and PYGL were obtained (Figure 3K), and their FoldChange is shown in Figure S5. Among them, BCL2, ESR1, FN1, and PYGL5 were significantly up-regulated, indicating that their regulatory roles in diverse biological processes, such as cell survival, hormone signaling, extracellular matrix remodeling, and glucose metabolism, are crucial for the successful establishment and maintenance of lactation.

### 3.6. Molecular Docking of HQ and Nine Core Targets

**Figure 4:**
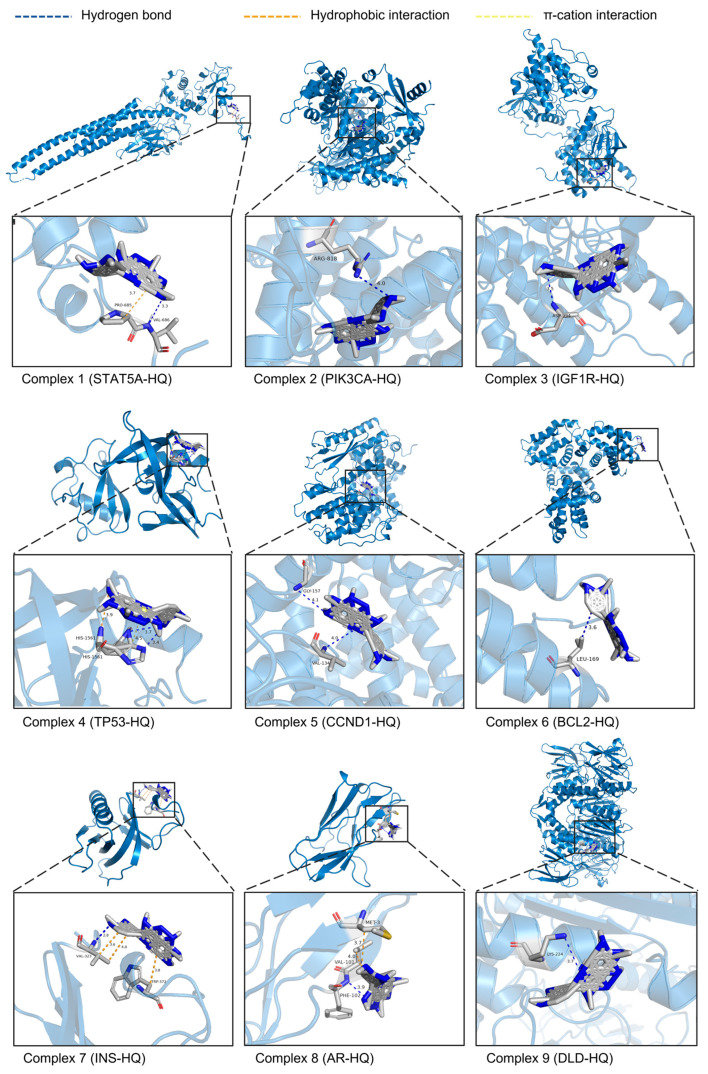
3D schematic diagram of the molecular docking complex between HQ and 9 potential core targets. The sky-blue structures represent the targets. Gray structures represent the target’s amino acid residues that bind to HQ. Blue dashed lines show the hydrogen bonds formed between HQ and the amino acid residues. Orange dashed lines show hydrophobic interactions. Yellow dashed lines show π-cation interactions. The numbers next to the interactions indicate their lengths in Å.

After the ITP network analysis, the key flavonoid component HQ and nine potential core targets (STAT5A, PIK3CA, IGF1R, TP53, CCND1, BCL2, INS, AR, and DLD) were obtained. A molecular docking study was performed on them using AutoDockTools 1.5.6 software, and docking patterns were visualized using PyMOL 2.6.0 software (Figure 4). The results showed that the binding energies of these nine groups of docking complexes were −4.35, −6.43, −4.99, −3.95, −3.92, −3.72, −3.97, −6.23, and −5.93 kcal/mol, respectively. Generally, binding energy with less than −4.25, −5.0, or −7.0 kcal/mol is considered as a certain good or strong binding activity between the ligand and the receptor, respectively. The docking complex formed by PIK3CA and HQ had the lowest binding energy, indicating the strongest affinity between them. The results showed that the ligand HQ can interact with PIK3CA by forming hydrogen bonds with residues Arg818A. The distance between the hydrogen atom and the acceptor atom is 3.45 Å, and the distance between the donor atom and the acceptor atom is 3.99 Å. The above results indicated that residue Arg818A plays an important role in the binding process of HQ and PIK3CA, and they also showed that the hydrogen bonding network is the primary factor contributing to the stability of this complex.

### 3.7. HQ Up-Regulated Lactation-Promoting Function in BMECs

**Figure 5:**
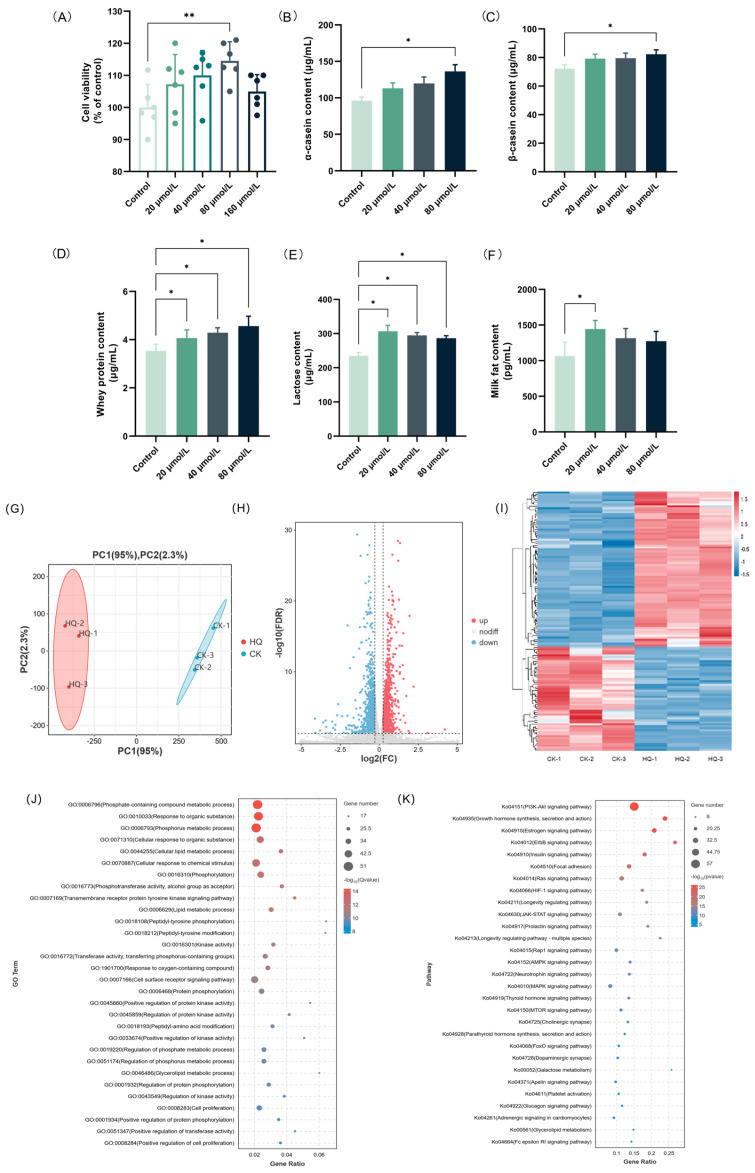
Effects of HQ treatment on lactation performance and transcriptional profile of BMECs. (**A**) The effect of various concentrations of HQ on the cell viability of BMECs. Data are mean ± SEM (*n* = 6). The contents of α-casein (**B**), β-casein (**C**), whey protein (**D**), lactose (**E**), and milk fat (**F**) of samples from the HQ group and the CK group. Data are mean ± SEM (*n* = 3); * *p* < 0.05, ** *p* < 0.01. (**G**) PCA diagram. The percentages indicate the contribution of each principal component to the variance in the dataset. (**H**) Volcano plot of DEGs. Genes expressed at higher levels are shown in red; genes expressed at lower levels are shown in blue. (**I**) Clustering heat map of DEGs. Hierarchical clustering is based on log_10_ (FPKM + 1). (**J**) GO enrichment analysis of DEGs. (**K**) KEGG enrichment analysis of DEGs.

The effects of the different concentrations of HQ (0–160 μM) on the cell viability of the BMECs were assessed using the CCK-8 assay (Figure 5A). The results indicated that all of the concentrations of HQ were non-toxic to BMEC. The effects of the different concentrations of HQ (0–80 μM) on the contents of milk proteins (α-casein, β-casein, and whey protein), lactose, and milk fat were investigated using the ELISA method. As shown in Figure 5B–D, HQ greatly increased the casein content in BMECs in a dose-dependent manner. In detail, the contents of α-casein, β-casein, and whey protein in the group treated with 80 μM HQ were 136.22, 82.33, and 4.56 μg/mL, respectively, which were 1.42, 1.14, and 1.29 times that of the CK group. In addition, the effects of HQ on the lactose and milk fat levels in BMECs are shown in Figure 5E,F. Our results indicate that, contrary to the results for milk proteins, low concentrations of HQ are more effective in stimulating the secretion of lactose and milk fat in BMECs. In detail, after treatment with 20 μM HQ, both the lactose and milk fat contents reached peak values of 306.85 μg/mL and 1443.85 pg/mL, respectively. The above results are consistent with the results of the HAE treatment of BMECs.

### 3.8. Effect of HQ on the Lactation-Related Pathways via DEG Enrichment Analysis

The transcriptome profiling was further used to investigate the potential mechanisms of the lactation-promoting effect of HQ (80 μM) in BMECs. The quality control and comparison results are shown in Tables S10 and S11, indicating high sequencing accuracy and reliable results. The effect of HQ on the transcript profile is shown in Figure 5G–I and Figure S6. Based on the differential analysis criteria of FoldChange > 1.2 and FDR < 0.05, a total of 1707 genes were differentially expressed, including 1172 up-regulated and 535 down-regulated genes. To narrow down the scope of the study, a total of 131 DEGs associated with the lactation process in the CK group vs. the HQ group were screened out (Table S12), and the GO and KEGG enrichment analyses of these DEGs were conducted. The GO enrichment analysis of DEGs related to the lactation process is shown in Figure 5J. These DEGs were mainly enriched in phosphate-containing compound metabolic process (GO:0006796), response to organic substance (GO:0010033), phosphorus metabolic process (GO:0006793), cellular response to organic substance (GO:0071310), and cellular lipid metabolic process (GO:0044255). It is shown that, similar to HAE, HQ also mainly acted on the phosphorus metabolism and lipid metabolism processes to promote the lactation performance of cells. The up-regulation of the genes involved in phosphorus metabolism and lipid metabolism may enhance energy production for milk synthesis and alter milk fat synthesis and composition. The KEGG enrichment analysis of the DEGs related to lactation process is shown in Figure 5K. These DEGs were mainly enriched in the PI3K-Akt signaling pathway (ko04151), growth hormone synthesis, secretion and action (ko04935), estrogen signaling pathway (ko04915), ErbB signaling pathway (ko04012), and insulin signaling pathway (ko04910). Among these metabolic pathways, the PI3K-Akt signaling pathway is still the metabolic pathway with the largest number of enriched DEGs and the highest significance, indicating that HQ may also exert lactation-promoting activity through the PI3K-Akt signaling pathway. The GO and KEGG enrichment results from the HQ-treated cells showed significant overlap with those observed in the HAE-treated cells, particularly in the metabolic and hormone-related signaling pathways. This similarity suggested that HQ may replicate the signaling pathways modulated by HAE in BMECs, thereby exerting a similar promotive effect on lactation function.

### 3.9. Analysis of Common DEGs of HAE and HQ

**Figure 6:**
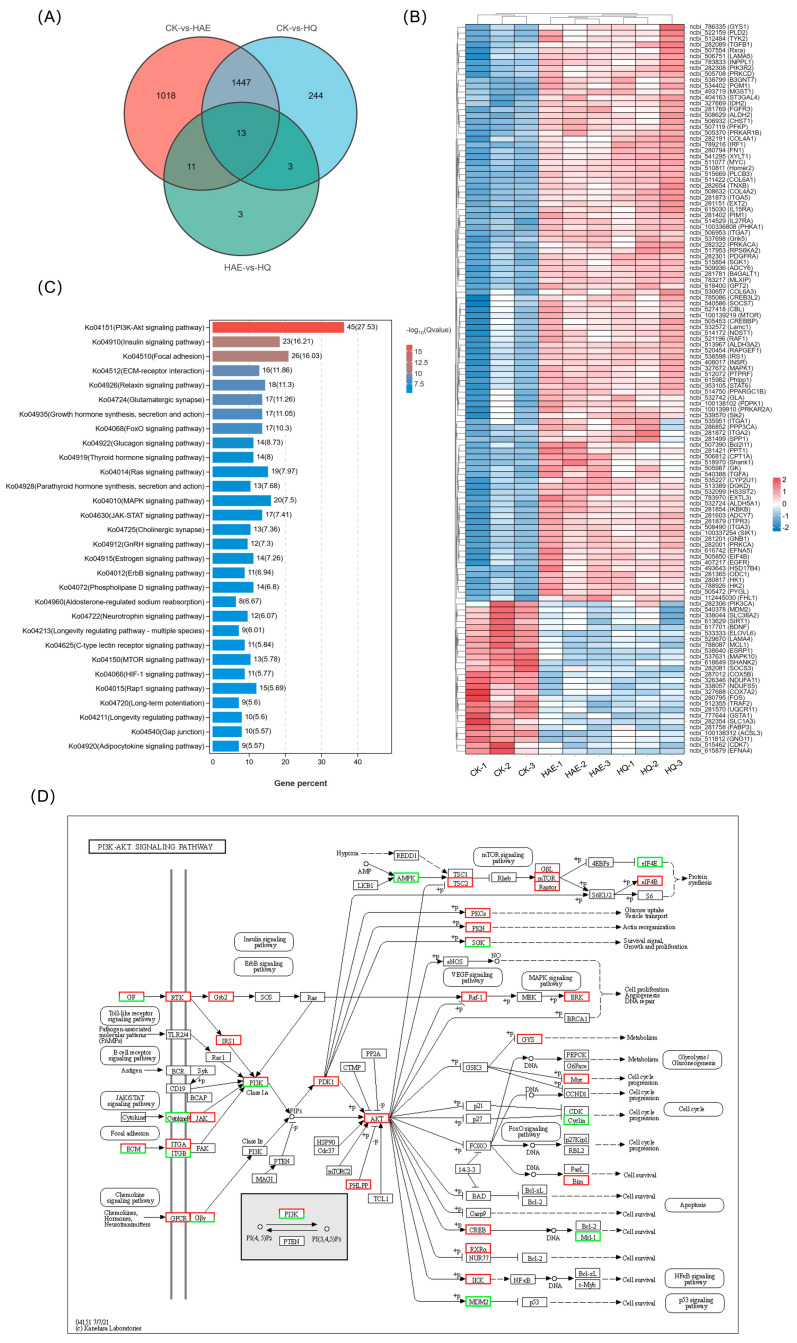

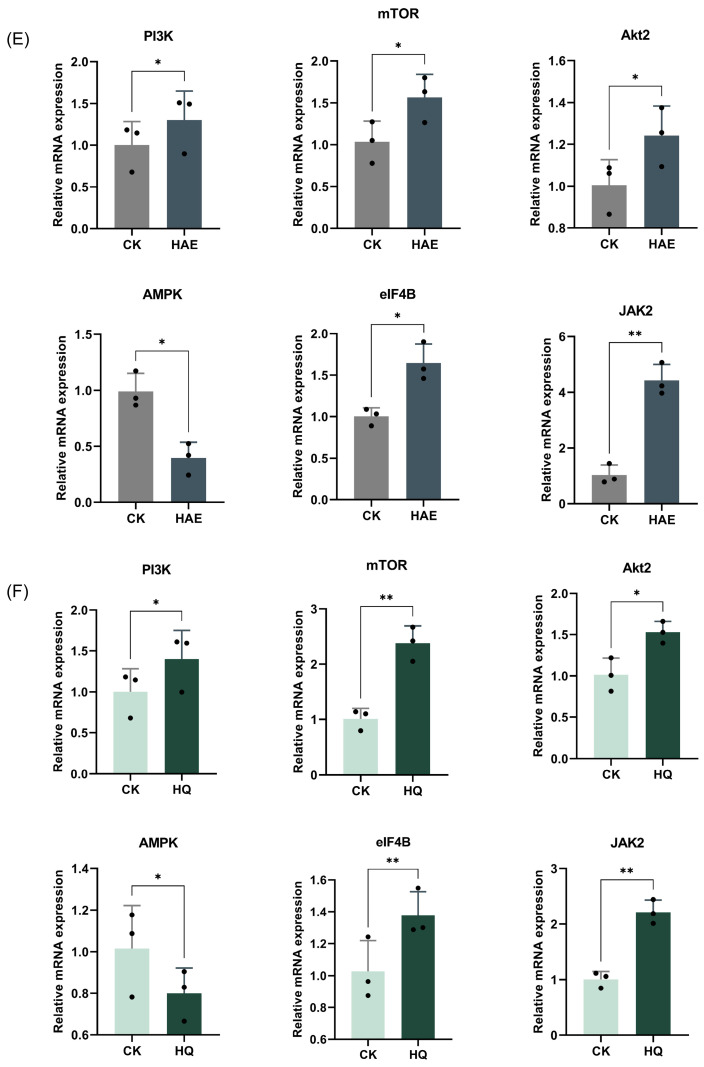
Analysis and RT-qPCR validation of common DEGs in CK vs. HAE and CK vs. HQ. (**A**) Venn diagram of common and unique DEGs among different comparison groups. (**B**) Correlation heat map of common DEGs in CK vs. HAE and CK vs. HQ related to lactation process. Redder squares indicate higher expression and bluer squares indicate lower expression. (**C**) KEGG enrichment of common DEGs in CK vs. HAE and CK vs. HQ related to lactation process. The abscissa indicates the percentage of lactation-related genes in the pathway to the total lactation-related genes. The color of the bars represents the −log_10_(Q value) of enrichment. (**D**) Effect of HAE and HQ on PI3K-Akt signaling pathway. Red means the DEGs are up-regulated, and green means down-regulated. Effect of 400 μg/mL HAE (**E**) and 80 μM HQ (**F**) on PI3K-Akt pathway via RT-qPCR assays. Data are mean ± SEM (*n* = 3); * *p* < 0.05, ** *p* < 0.01.

In order to investigate the lactation-promoting mechanism of HQ and its relationship with HAE, the expression changes of common DEGs in CK vs. HAE and CK vs. HQ were investigated. As shown in Figure 6A, the Venn diagram revealed 1460 common DEGs between CK vs. HAE and CK vs. HQ. From them, 125 DEGs were selectively screened based on lactation-related information, and the expression levels of these common DEGs were further analyzed using a correlation heat map (Figure 6B). The results showed that the genes involved in milk protein synthesis, including *mTOR* (ncbi_100139219), *EIF4B* (ncbi_505850), *INSR* (ncbi_408017), *PRKACA* (ncbi_282322), *PRKAR1B* (ncbi_505370), and *PRKAR2A* (ncbi_100139910), were up-regulated. Additionally, the genes involved in lactose synthesis, involving *GYS1* (ncbi_786335), *PGM1* (ncbi_534402), *PRKACA* (ncbi_282322), and *B4GALT1* (ncbi_281781), were up-regulated. Furthermore, an increase in the expression of the genes related to milk fat synthesis was observed, including *GK* (ncbi_505987) and *PPARGC1B* (ncbi_514750). The KEGG enrichment analysis of these common DEGs in CK vs. HAE and CK vs. HQ related to the lactation process was performed (Figure 6C). The results showed that 45 of these DEGs were enriched in the PI3K-Akt signaling pathway (ko04151), 23 DEGs were enriched in the insulin signaling pathway (ko04910), and 26 DEGs were enriched in focal adhesion (ko04510). This finding further confirmed that the PI3K-Akt signaling pathway is the most critical metabolic pathway for HAE and HQ to exert their lactation-promoting effects, and the effects of HAE and HQ on the PI3K-Akt signaling pathway are shown in Figure 6D. The results showed that 38 lactation-related DEGs were up-regulated and 7 lactation-related DEGs were down-regulated in the PI3K-Akt signaling pathway.

### 3.10. Effect of HAE and HQ on PI3K-Akt Pathway via RT-qPCR Assays

To confirm the gene expression patterns identified at the transcriptome level, six key genes associated with the PI3K-Akt signaling pathway were analyzed for RT-qPCR validation (Figure 6E,F). RT-qPCR is a highly sensitive technique that quantifies mRNA levels, providing a reliable measure of gene expression, and the 2^−△△Ct^ method was used to calculate the relative changes in gene expression. The results showed that the mRNA expression levels of *PI3K*, *Akt2*, *mTOR*, *JAK2*, and *eIF4B* were significantly up-regulated, while *AMPK* was down-regulated after the HAE or HQ treatment (*p* < 0.05). The up-regulation of *PI3K*, *Akt2*, *mTOR*, *JAK2*, and *eIF4B* suggested an enhanced activation of the PI3K-Akt signaling pathway, which is known to play an important role in cell growth, proliferation, and lactation. Conversely, the down-regulation of *AMPK*, a gene associated with the energy regulation and inhibition of anabolic processes, supported the shift towards promoting lactation. The results confirmed that qPCR analysis was consistent with the RNA-seq data, further validating the accuracy of the transcriptomic. Additionally, these results revealed that both HAQ and HQ exhibited similar trends in the modulation of key genes in the PI3K-Akt pathway.

## 4. Discussion

Recognized in the ancient traditional Chinese medicine books, *H. citrina* has been historically employed to promote milk secretion in lactating women [22]. However, modern research has not provided clear evidence to clarify this historical claim. In this study, the lactation-promoting effects of *H. citrina* on BMECs and lactating rats are verified. The results showed that HAE significantly (*p* < 0.05) increased the synthesis of milk proteins, lactose, and fat in BMECs and enhanced the weight gain of pups, as well as the mammary gland tissue indices, uterine indices, and serum hormone levels (PRL, PRLR, and E_2_) of lactating rats. Additionally, HAE was observed to stimulate mammary gland development, accelerate the transformation of the lobular acinar system, enhance acinar density and secretion, and reduce fibrous connective and fatty tissues. It was revealed that PRL, PRLR, and E_2_ are essential in the development of the mammary gland, the initiation of milk production, and the maintenance of milk secretion [23]. During pregnancy, PRL directly or indirectly promotes the development of milk-secreting lobuloalveolar by binding to PRLR or regulating the systemic hormonal environment through the pituitary–ovarian axis [24]. Oakes et al. [24] noted that PRL can induce ductal side branching by regulating the production of ovarian progesterone. During lactation, PRL modulates mammary gland development and milk production by activating the JAK2-STAT5 signaling pathway [25]. Upon binding to PRLR on the cell membrane, PRL stimulates JAK2, which phosphorylates the intracellular tyrosines of receptor complex after receptor oligomerization [26]. This progress creates docking sites for STAT5. The phosphorylated STAT5A and STAT5B form homodimers, and heterodimers then migrate to the cell nucleus in mammary epithelial cells to initiate the transcription of casein genes, thus promoting lactation. Zhou et al. [27] demonstrated that PRL is capable of regulating the expression and activity of the L-type amino acid transporter 1 (LAT1) in mammary epithelial cells through the STAT5 pathway. This regulation enhanced the availability of amino acids and the synthesis of milk protein in the mammary glands of dairy cows. Estrogen, specifically E_2_, the most potent form of mammalian estrogen, is crucial for ductal development and mammary epithelial cell proliferation. Arendt and Kuperwasser [28] reported that E_2_ can drive the rapid growth of ducts into the mammary fat pad through its receptor α (ERα). Furthermore, Błasiak and Molik [29] found that E_2_ can indirectly prompt the pituitary gland to release PRL and to increase the presence of PRLR in the mammary gland.

To systematically reveal the key components of the lactation-promoting effect in *H. citrina*, water (the safest and most frequently used food-grade solvent) was utilized to extract the functional components in HAE. In this study, a total of 499 components were identified through UHPLC-OE-MS analysis. The predominant constituents included flavonoids, saccharides and alcohols, lipids, amino acids and derivatives, and phenolics. Consistent with our findings, recent research utilizing UPLC-MS/MS identified a total of 728 metabolites within *H. citrina*, and flavonoids, lipids, phenolic acids, and amino acids, and their derivatives, were found to be the main components [10]. Ma et al. [9] determined the components of *H. citrina* using UHPLC-Q-TOF-MS/MS and UHPLC-QQQ-MS/MS analysis and identified 132 components, encompassing flavonoids, phenylpropanoids, lipids, alkaloids, and other types of compounds. In these compounds, flavonoids represent a class of secondary metabolites widely present in nature. Numerous studies have demonstrated that plant flavonoids contribute to improving lactation performance [30,31]. In our analysis of the flavonoid composition in *H. citrina*, HQ, 4′,5,6,7,8-pentahydroxy-3’-methoxyflavone, rutin, myricetin 3-robinobioside, licoisoflavone A, myricetin 3-galactoside, naringenin, quercetin, and kaempferol were identified as the primary flavonoid compounds. Among them, rutin, kaempferol, quercetin, and naringenin have been well documented for their beneficial impacts on mammary health and milk production enhancement [17,19,32]. However, the lactogenic potential of HQ, the most abundant flavonoid component in our study, has not been previously reported.

The data from the current study indicate that the cell proliferation and synthesis of milk proteins, lactose, and fat in BMECs were positively regulated with the treatment of HQ, indicating that HQ has a good lactation-promoting effect and may be the potential material basis for the lactation-promoting activity of HAE. Transcriptomic coupled with RT-qPCR further revealed that DEGs in both the HAE and HQ groups were mainly enriched in the PI3K-Akt signaling pathway, indicating that HAE and HQ primarily exert their lactogenic effects through the PI3K-Akt signaling pathway. It is noteworthy that, although we have demonstrated that HQ can promote lactation by regulating the same metabolic pathways as HAE, the synergistic effects between different compounds should not be ignored. For instance, Shalev et al. [33] found that extracts of *Pistacia lentiscus* (lentisk) were more effective in enhancing mitochondrial productivity and activity and regulating the secretion of milk components compared to isolated active components like myricetin or gallic acid, indicating that different active components have additive or synergistic effects on the lactation performance of mammary epithelial cells.

To clarify the underlying mechanisms of the lactation-promoting effect, common DEGs related to lactation between CK and HAE and CK and HQ were screened, and the functional enrichment analysis of these DEGs was performed. The results showed that these DEGs were indeed mainly enriched in the PI3K-Akt signaling pathway, and the mRNA expression of genes in the PI3K-Akt signaling pathway, including *EGFR*, *FGFR3*, *INSR*, *IRS1*, *PI3K*, *GNB1*, *GNG4*, *Akt2*, *mTOR*, *eIF4B*, *MYC*, *PRKCA*, and *PRKAR1B*, were significantly up-regulated in comparison to the CK group in both the HAE and HQ-treated groups (*p* < 0.05).

The proteins in milk mainly include casein, whey protein, various enzymes and endogenous peptides, etc., which provide basic nutritional support for the growth and development of infants [34]. In the PI3K-Akt signaling pathway, signaling molecules bind to corresponding receptors such as the epidermal growth factor receptor (EGFR), fibroblast growth factor receptor 3 (FGFR3), and insulin receptor (INSR) to activate genes encoding downstream factors, namely, insulin receptor substrate-1 (IRS1), G protein subunit beta 1 (GNB1), and G protein subunit gamma 4 (GNG4) [35]. Then, activated phosphoinositide 3-kinase (PI3K) induces the conversion of phosphatidylinositol (4,5)-bisphosphate (PIP2) into phosphatidylinositol (3,4,5)-trisphosphate (PIP3) through a cascade reaction, further activating protein kinase B (Akt) [36]. This resulted in the up-regulation of mTOR and the subsequent induction of eukaryotic initiation factor 4B (eIF4B) expression, thereby enhancing translation initiation and promoting protein synthesis. Previous research has reported that mTOR contributed to regulating the mRNA levels for *CSN1S1*, *CSN2* and *CSN3*, encoding αs1-casein, β-casein, and κ-casein, respectively, in BMECs via 4E binding protein (4EBP1) and ribosomal protein S6 kinase 1 (S6K1) [37,38].

Additionally, research indicated that 55–70% of glucose in the mammary gland is utilized for lactose synthesis, and a portion of glucose serves as a substrate to power the synthesis of lactose [39]. In this study, HAE and HQ treatment significantly increased the expression of *PRKCA*, *GYS*, *PGM1*, and *B4GALT1*. In the PI3K-Akt signaling pathway, up-regulated PI3K activates downstream effectors such as phosphoinositide-dependent kinase-1 (PDPK1) and Akt through a cascade reaction. Activated PDK1 further phosphorylates protein kinase C α (*PRKCA*), enhances glucose uptake and intracellular vesicle transport, and provides raw materials and energy for lactose synthesis [40]. At the same time, the activation of Akt increases the activity of glycogen synthase (GYS) and promotes glycogen synthesis. The synthesized glycogen then participates in galactose metabolism and lactose synthesis processes, respectively, under the action of up-regulated genes *PGM1* and *B4GALT1* in other pathways. Similarly, Sevrin et al. [41] found that dietary fenugreek supplementation can promote the synthesis of milk components by up-regulating the expression of genes related to the uptake of glucose (*GLUT1*), metabolism of galactose (*PGM1*), and synthesis of lactose (*B4GALT1*).

**Figure 7:**
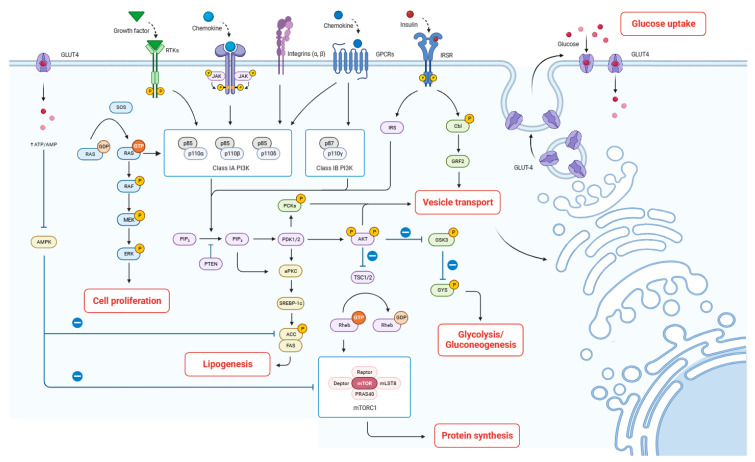
Proposed mechanism by which HAE improves lactation. The arrows indicate the direction of signal transduction or molecular interactions. Solid arrows represent activation or stimulation of downstream molecules or pathways. Dashed lines with a bar at the end indicate inhibition or suppression of downstream targets.

Milk fat synthesis includes fatty acid de novo synthesis, uptake, activation, intracellular trafficking, elongation, desaturation, triacylglycerol assembly, and lipid droplet formation within the mammary gland epithelial cells [37]. This study revealed that the mRNA expression of *PI3K*, mTOR in the PI3K-Akt signaling pathway, and *GK* in the insulin signaling pathway were significantly up-regulated. The enhanced expression of *PI3K* and *mTOR* plays a key role in promoting fatty acid synthesis and subsequent lipid droplet formation in mammary gland epithelial cells. This up-regulation facilitates anabolic processes, including the synthesis of triacylglycerols, the primary constituents of milk fat. Wang et al. [42] also found that acylated ghrelin promotes milk fat synthesis in BMECs through the PI3K-mTOR signaling pathway. Additionally, the up-regulation of *GK* within the insulin signaling pathway further promotes its conversion to glycerol-3-phosphate, a backbone for triacylglycerol formation [43]. Taken together, we speculate that HAE and HQ could improve lactogenic activity by enhancing milk protein synthesis, regulating glucose uptake and galactose metabolism, and promoting fatty acid synthesis (Figure 7).

## 5. Conclusions

In conclusion, our study provides systematic evidence supporting the lactation-promoting effect of the flavonoid compounds in HAE in lactating rats and BMECs. Network pharmacology predicted that HQ, cichoriin, paeonoside, jaceidin, and catechin were possible lactogenic active components, and STAT5A, PIK3CA, IGF1R, TP53, and CCND1 were potential core targets. HQ was identified for the first time as the flavonoid with the highest concentration in HAE. RNA-seq studies further revealed the importance of the PI3K-Akt signaling pathway. Cell-based experiments combined with qRT-PCR verified the potential pathways and its molecular targets. The analysis of common DEGs in CK vs. HAE and CK vs. HQ indicated that both HAE and HQ enhanced lactogenic activity mainly through the PI3K-Akt signaling pathway by improving milk protein synthesis, regulating glucose uptake and galactose metabolism, and promoting fatty acid synthesis. However, the lactation-promoting potential of HQ was only evaluated at the cellular level in this study. Further validation in animal models is necessary to confirm its efficacy and safety in a more complex biological system. Additionally, more comprehensive and systematic studies such as multi-omics analysis, deep machine learning, and single-cell sequencing to verify its biological effects and underlying mechanisms should be needed in the future. These results provide scientific evidence for HQ as a functional food factor in *H. citrina* that promotes lactation.

## Figures and Tables

**Table 1:** Composition and identification of top 20 flavonoids in HAE.

Compound Name	Formula	Molecular Weight	CAS	*m*/*z*	Ionization Model	Peak Area
Hexamethylquercetagetin	C_21_H_22_O_8_	402.4	1251-84-9	401.13	[M − H]−	421,194,790.80
4′,5,6,7,8-Pentahydroxy-3’-methoxyflavone	C_16_H_12_O_8_	332.26	181020-34-8	333.07	[M + H]+	64,114,119.42
Rutin	C_27_H_30_O_16_	610.5	153-18-4	611.16	[M + H]+	60,833,174.93
Myricetin 3-robinobioside	C_27_H_30_O_17_	626.5	145544-43-0	627.15	[M + H]+	59,991,262.61
Licoisoflavone A	C_20_H_18_O_6_	354.4	66056-19-7	353.11	[M − H]−	59,916,514.78
Myricetin 3-galactoside	C_21_H_20_O_13_	480.4	15648-86-9	481.10	[M + H]+	35,915,744.05
Naringenin	C_15_H_12_O_5_	272.25	480-41-1	271.06	[M − H]−	18,411,397.14
Quercetin	C_15_H_10_O_7_	302.23	117-39-5	301.03	[M − H]−	15,911,669.73
Kaempferol	C_15_H_10_O_6_	286.24	520-18-3	287.05	[M + H]+	14,296,488.93
7-Hydroxy-2-methylisoflavone	C_16_H_12_O_3_	252.26	2859-88-3	253.08	[M + H]+	14,280,814.31
Petunidin 3-galactoside	C_22_H_23_O_12+_	479.4	28500-02-9	479.12	[M + H]+	14,378,042.42
Daidzin	C_21_H_20_O_9_	416.4	552-66-9	415.11	[M − H]−	7,576,634.70
Lutein	C_40_H_56_O_2_	568.9	127-40-2	568.43	[M + H]+	10,175,254.17
Malvidin 3-(6-acetylglucoside)	C_25_H_27_O_13+_	535.5	101072-66-6	536.16	[M + H]+	10,822,103.81
Orientin	C_21_H_20_O_11_	448.4	28608-75-5	449.11	[M + H]+	5,787,667.10
Multinoside A	C_27_H_30_O_16_	610.5	59262-54-3	645.12	[M − H]−	6,037,243.24
Isorhamnetin	C_16_H_12_O_7_	316.26	480-19-3	317.06	[M + H]+	4,581,085.32
Myricetin 3-arabinoside	C_20_H_18_O_12_	450.3	132679-85-7	451.09	[M + H]+	4,635,990.00
Citroside A	C_19_H_30_O_8_	386.4	120330-44-1	387.20	[M + H]+	3,243,535.37
4′,8-Dimethylgossypetin 3-glucoside	C_23_H_24_O_13_	508.4	90456-57-8	509.13	[M + H]+	3,126,823.92

## Data Availability

The original contributions presented in the study are included in the article/Supplementary Materials, further inquiries can be directed to the corresponding author.

## Supplementary Materials

The following supporting information can be downloaded at: https://www.mdpi.com/article/10.3390/foods13172813/s1. Figure S1: All DEGs of GO and KEGG enrichment analyses treated with HAE (400 μg/mL); Figure S2: The total ion current (TIC) diagrams of compounds in HAE by UHPLC-Q-Exactive Orbitrap-MS; Figure S3: PPI network information obtained from STRING12.0 database; Figure S4: GO (A) and KEGG (B) enrichment analyses of the intersection of potential targets for HAE flavonoids and lactation-related targets; Figure S5: 5 key genes shared by network pharmacology and lactation-related DEGs and their FoldChange; Figure S6: Violin plot for gene expression. The *x*-axis of the plot represents different samples; the *y*-axis represents the logarithmic scale of FPKM expression levels for the samples; Table S1: List of RT-qPCR primer sequences used; Table S2: Quality control of all sample summary in the CK group vs. the HAE group; Table S3: Comparison results of sequencing reads and reference genome in the CK group vs. the HAE group; Table S4: Description of DEGs related to the lactation process in the CK group vs. the HAE group; Table S5: The intersection of potential targets for HAE flavonoids and lactation-related targets; Table S6: KEGG enrichment analysis of the intersection of potential targets for HAE flavonoids and lactation-related targets; Table S7: KEGG enrichment analysis of the potential core targets; Table S8: Topological parameters of the potential core flavonoids in the ITP network; Table S9: Topological parameters of the potential core targets in the ITP network; Table S10: Quality control of all sample summary in the CK group vs. the HQ group; Table S11: Comparison results of sequencing reads and reference genome in the CK group vs. the HQ group; Table S12: Description of DEGs related to the lactation process in the CK group vs. the HQ group.

## References

[1] Kent J.C., Ashton E., Hardwick C.M., Rea A., Murray K., Geddes D.T. (2021). Causes of perception of insufficient milk supply in Western Australian mothers. Matern. Child Nutr..

[2] Gianni M.L., Bettinelli M.E., Manfra P., Sorrentino G., Bezze E., Plevani L., Cavallaro G., Raffaeli G., Crippa B.L., Colombo L. (2019). Breastfeeding difficulties and risk for early breastfeeding cessation. Nutrients.

[3] Li D., Li Y., Chen Y., Li H., She Y., Zhang X., Chen S., Chen W., Qiu G., Huang H. (2019). Neuroprotection of reduced thyroid hormone with increased estrogen and progestogen in postpartum depression. Biosci. Rep..

[4] Grzeskowiak L.E., Wlodek M.E., Geddes D.T. (2019). What evidence do we have for pharmaceutical galactagogues in the treatment of lactation insufficiency?—A narrative review. Nutrients.

[5] Shen Q., Khan K.S., Du M.C., Du W.W., Ouyang Y.Q. (2021). Efficacy and safety of domperidone and metoclopramide in breastfeeding: A systematic review and meta-analysis. Breastfeed. Med..

[6] Li C., Dai T., Chen J., Chen M., Liang R., Liu C., Du L., McClements D.J. (2023). Modification of flavonoids: Methods and influences on biological activities. Crit. Rev. Food Sci. Nutr..

[7] Liu J., Zhong X., Jiang Y., Yu L., Huang X., Dong Z., Yang S., He W., Zeng J., Qing Z. (2020). Systematic identification metabolites of *Hemerocallis citrina* Borani by high-performance liquid chromatography/quadrupole-time-of-flight mass spectrometry combined with a screening method. J. Pharm. Biomed. Anal..

[8] Li C.F., Chen X.Q., Chen S.M., Chen X.M., Geng D., Liu Q., Yi L.T. (2017). Evaluation of the toxicological properties and anti-inflammatory mechanism of *Hemerocallis citrina* in LPS-induced depressive-like mice. Biomed. Pharmacother..

[9] Ma T., Lin J., Gan A., Sun Y., Sun Y., Wang M., Wan M., Yan T., Jia Y. (2023). Qualitative and quantitative analysis of the components in flowers of *Hemerocallis citrina* Baroni by UHPLC–Q-TOF-MS/MS and UHPLC–QQQ-MS/MS and evaluation of their antioxidant activities. J. Food Compos. Anal..

[10] Liang Y., Zhan X., Wei X., Zhong J., Deng J., Chen Y., Pan L., Zhang J., Li M., Huang R. (2023). Integration of omics and targeted screening strategy provide insights into the material basis and mechanism of *Hemerocallis citrina* Baroni on sleep-improvement. Food Res. Int..

[11] Zhao R., Luo J., Xu B. (2024). Insights into secondary metabolites and health promoting effects of edible flower *Hemerocallis citrina* Baroni. J. Funct. Foods.

[12] Zhong J., Liang Y., Chen Y., Zhang J., Zou X., Deng J., Wang D., Sun Y., Li M. (2021). Study and experimental validation of the functional components and mechanisms of *Hemerocallis citrina* Baroni in the treatment of lactation deficiency. Foods.

[13] Guo S., Qin N., Wang X., Zuo Z., Li Q., Wang Y. (2023). Freeze-dried powder of daylily bud improves bromocriptine-induced lactation disorder in rats via JAK2/STAT5 pathway. J. Ethnopharmacol..

[14] Shen N., Wang T., Gan Q., Liu S., Wang L., Jin B. (2022). Plant flavonoids: Classification, distribution, biosynthesis, and antioxidant activity. Food Chem..

[15] Wen K., Fang X., Yang J., Yao Y., Nandakumar K.S., Salem M.L., Cheng K. (2021). Recent research on flavonoids and their biomedical applications. Curr. Med. Chem..

[16] Garavaglia L., Galletti S., Tedesco D. (2015). Silymarin and lycopene administration in periparturient dairy cows: Effects on milk production and oxidative status. New Zealand Vet. J..

[17] Cui K., Guo X., Tu Y., Zhang N., Ma T., Diao Q. (2015). Effect of dietary supplementation of rutin on lactation performance, ruminal fermentation and metabolism in dairy cows. J. Anim. Physiol. Anim. Nutr..

[18] Abdou R.M., Fathey M. (2018). Evaluation of early postpartum fenugreek supplementation on expressed breast milk volume and prolactin levels variation. Egypt. Pediatr. Assoc..

[19] Lin M., Wang N., Yao B., Zhong Y., Lin Y., You T. (2018). Quercetin improves postpartum hypogalactia in milk-deficient mice via stimulating prolactin production in pituitary gland. Phytother. Res..

[20] Ahamad N., Sun Y., Nascimento Da Conceicao V., Xavier Paul Ezhilan C.R., Natarajan M., Singh B.B. (2021). Differential activation of Ca2+ influx channels modulate stem cell potency, their proliferation/viability and tissue regeneration. Npj Regener. Med..

[21] Guo Z., Yin H., Wu T., Wu S., Liu L., Zhang L., He Y., Zhang R., Liu N. (2022). Study on the mechanism of Cortex Lycii on lung cancer based on network pharmacology combined with experimental validation. J. Ethnopharmacol..

[22] Liang Y., Wei X., Ren R., Zhang X., Tang X., Yang J., Wei X., Huang R., Hardiman G., Sun Y. (2023). Study on anti-constipation effects of *Hemerocallis citrina* Baroni through a novel strategy of network pharmacology screening. Int. J. Mol. Sci..

[23] Ni Y., Chen Q., Cai J., Xiao L., Zhang J. (2021). Three lactation-related hormones: Regulation of hypothalamus-pituitary axis and function on lactation. Mol. Cell. Endocrinol..

[24] Oakes S.R., Rogers R.L., Naylor M.J., Ormandy C.J. (2008). Prolactin regulation of mammary gland development. J. Mammary Gland Biol. Neoplasia.

[25] Khan M.Z., Khan A., Xiao J., Ma Y., Ma J., Gao J., Cao Z. (2020). Role of the JAK-STAT pathway in bovine mastitis and milk production. Animals.

[26] Hannan F.M., Elajnaf T., Vandenberg L.N., Kennedy S.H., Thakker R.V. (2023). Hormonal regulation of mammary gland development and lactation. Nat. Rev. Endocrinol..

[27] Zhou J., Jiang M., Shi Y., Song S., Hou X., Lin Y. (2020). Prolactin regulates LAT1 expression via STAT5 (signal transducer and activator of transcription 5) signaling in mammary epithelial cells of dairy cows. J. Dairy Sci..

[28] Arendt L.M., Kuperwasser C. (2015). Form and function: How estrogen and progesterone regulate the mammary epithelial hierarchy. J. Mammary Gland Biol. Neoplasia.

[29] Błasiak M., Molik E. (2015). Role of hormones and growth factors in initiating and maintaining the lactation of seasonal animals. Med. Weter.

[30] Hassan F.U., Arshad M.A., Li M., Rehman M.S.U., Loor J.J., Huang J. (2020). Potential of mulberry leaf biomass and its flavonoids to improve production and health in ruminants: Mechanistic insights and prospects. Animals.

[31] Olagaray K., Bradford B. (2019). Plant flavonoids to improve productivity of ruminants—A review. Anim. Feed Sci. Technol..

[32] Simitzis P., Massouras T., Goliomytis M., Charismiadou M., Moschou K., Economou C., Papadedes V., Lepesioti S., Deligeorgis S. (2019). The effects of hesperidin or naringin dietary supplementation on the milk properties of dairy ewes. J. Sci. Food Agric..

[33] Shalev Y., Hadaya O., Bransi-Nicola R., Landau S.Y., Azaizeh H., Muklada H., Glasser T., Roth Z., Deutch-Traubman T., Haj-Zaroubi M. (2022). Entourage effect for phenolic compounds on production and metabolism of mammary epithelial cells. Heliyon.

[34] Auestad N., Layman D.K. (2021). Dairy bioactive proteins and peptides: A narrative review. Nutr. Rev..

[35] Nandi S., Dey R., Samadder A., Saxena A., Saxena A.K. (2022). Natural sourced inhibitors of EGFR, PDGFR, FGFR and VEGFR-mediated signaling pathways as potential anticancer agents. Curr. Med. Chem..

[36] Tariq K., Luikart B.W. (2021). Striking a balance: PIP2 and PIP3 signaling in neuronal health and disease. Explor. Neuroprot. Ther..

[37] Wu Z., Tian M., Heng J., Chen J., Chen F., Guan W., Zhang S. (2020). Current evidences and future perspectives for AMPK in the regulation of milk production and mammary gland biology. Front. Cell Dev. Biol..

[38] Zhang M., Zhao S., Wang S., Luo C., Gao H., Zheng N., Wang J. (2018). d-Glucose and amino acid deficiency inhibits casein synthesis through JAK2/STAT5 and AMPK/mTOR signaling pathways in mammary epithelial cells of dairy cows. J. Dairy Sci..

[39] Myronyuk I.F., Mandzyuk V.I., Sachko V.M., Gun’ko V.M. (2016). Structural features of carbons produced using glucose, lactose, and saccharose. Nanoscale Res. Lett..

[40] Lee E.E., Ma J., Sacharidou A., Mi W., Salato V.K., Nguyen N., Jiang Y., Pascual J.M., North P.E., Shaul P.W. (2015). A protein kinase C phosphorylation motif in GLUT1 affects glucose transport and is mutated in GLUT1 deficiency syndrome. Mol. Cell.

[41] Sevrin T., Boquien C.Y., Gandon A., Grit I., de Coppet P., Darmaun D., Alexandre-Gouabau M.-C. (2020). Fenugreek stimulates the expression of genes involved in milk synthesis and milk flow through modulation of insulin/GH/IGF-1 axis and oxytocin secretion. Genes.

[42] Wang J., Cao Y., Long X., Li F., Jiang N., Sun M., Xie Y., Ge Y., Guo W., Liu J. (2023). Acylated Ghrelin Activates PI3K/mTOR Signaling Pathway by Promoting ThPOK Acetylation to Promote Milk Fat Synthesis in Bovine Mammary Epithelial Cells. J. Agric. Food Chem..

[43] Li N., Zhao F., Wei C., Liang M., Zhang N., Wang C., Li Q.Z., Gao X.J. (2014). Function of SREBP1 in the milk fat synthesis of dairy cow mammary epithelial cells. Int. J. Mol. Sci..

