# Supplementary material for: The Aqueous Extract of Hemerocallis citrina Baroni Improves the Lactation-Promoting Effect in Bovine Mammary Epithelial Cells through the PI3K-AKT Signaling Pathway
Source: Foods. 2024 Sep 4;13(17):2813. doi: 10.3390/foods13172813 (PMC11395325; doi:10.3390/foods13172813)
Supplement: Supplementary file 1 [file foods-13-02813-s001.zip › foods-3101453-supplementary.pdf]

**Supplementary Materials**

**Table caption**

**Table S1** List of RT-qPCR primer sequences used.

**Table S2** Quality control of all sample summary in the CK group vs the HAE group.

**Table S3** Comparison results of sequencing reads and reference genome in the CK group vs the HAE group.

**Table S4** Description of DEGs related to the lactation process in the CK group vs the HAE group.

**Table S5** The intersection of potential targets for HAE flavonoids and lactation-related targets.

**Table S6** KEGG enrichment analysis of the intersection of potential targets for HAE flavonoids and lactation-related targets.

**Table S7** KEGG enrichment analysis of the potential core targets.

**Table S8** Topological parameters of the potential core flavonoids in the ITP network.

**Table S9** Topological parameters of the potential core targets in the ITP network.

**Table S10** Quality control of all sample summary in the CK group vs the HQ group.

**Table S11** Comparison results of sequencing reads and reference genome in the CK group vs the HQ group.

**Table S12** Description of DEGs related to the lactation process in the CK group vs the HQ group.

**Figure captions**

**Fig. S1** All DEGs of GO and KEGG enrichment analysis treated with HAE (400 μg/mL). (A) GO enrichment analysis. The up-regulated genes in the HAE group are represented by blue bars and the downregulated genes are represented by red bars. (B) KEGG enrichment analysis. The size of the circles corresponds to the quantity of DEGs enriched within each pathway. the color of the circles represents the $-\log_{10}$(Q value) of the enrichment significance.

**Fig. S2** The total ion current (TIC) diagrams of compounds in HAE by UHPLC-Q-Exactive Orbitrap-MS in (A) LC-MS (ESI+) and (B) LC-MS (ESI−).

**Fig. S3** PPI network information obtained from STRING12.0 database.

**Fig. S4** GO (A) and KEGG (B) enrichment analysis of the intersection of potential targets for HAE flavonoids and lactation-related targets.

**Fig. S5** 5 key genes shared by network pharmacology and lactation-related DEGs and their FoldChange.

**Fig. S6** Violin plot for gene expression. The x-axis of the plot represents different samples; the y-axis represents the logarithmic scale of FPKM expression levels for the samples.

**Table S1.** List of RT-qPCR primer sequences used.

| Gene name | Forward primer | Reverse primer |
|---|---|---|
| *GAPDH* | GGGTCATCATCTCTGCACCT | GGTCATAAGTCCCTCCACGA |
| *PI3KR1* | ACCATTCTTGGTTGCTGAAACTC | CACCTTGGTGTTTGGATTTCTG |
| *AMPK* | TTACTTGGCAACGAGCCCAC | CCCTGGGAGTTTCAGCAAC |
| *mTOR* | TGTGGAGTTTGAGGTGAAGC | ATTATCAAAGAAGGGCTGCAC |
| *JAK2* | AGATGGGAAGGGAAGGGTGTGG | AAGAGGGAGCAGCACGAAGGAT |
| *Akt2* | CCTGCTCTCTGGGCTACTCAA | CACGATGCTGGCGAAGAA |
| *eIF4B* | AGCTGGCGAAGTGAAGAAAC | GGCAAAGCCTGTTGACACAGCT |

**Table S2.** Quality control of all sample summary in the CK group vs the HAE group.

| Sample | RawReads | RawBases(G) | CleanReads | CleanBases(G) | Q30(%) | GC(%) |
|---|---|---|---|---|---|---|
| CK-1 | 48261916 | 7.24 | 48002428 | 7.17 | 92.81% | 51.56% |
| CK-2 | 48438562 | 7.27 | 48184804 | 7.2 | 93.00% | 51.68% |
| CK-3 | 48515642 | 7.28 | 48223154 | 7.22 | 92.35% | 52.03% |
| HAE-1 | 49195048 | 7.38 | 48867836 | 7.30 | 91.53% | 52.26% |
| HAE-2 | 48944278 | 7.34 | 48643228 | 7.27 | 92.12% | 52.16% |
| HAE-3 | 47487148 | 7.12 | 47134852 | 7.06 | 90.86% | 52.18% |

**Table S3.** Comparison results of sequencing reads and reference genome in the CK group vs the HAE group.

| Sample | Unmapped(%) | Unique Mapped(%) | Multiple Mapped(%) | Total Mapped(%) |
|---|---|---|---|---|
| CK-1 | 1341940 (2.81%) | 45152859 (94.57%) | 1250219 (2.62%) | 46403078 (97.19%) |
| CK-2 | 1397362 (2.91%) | 45363928 (94.57%) | 1209788 (2.52%) | 46573716 (97.09%) |
| CK-3 | 1501961 (3.13%) | 45292686 (94.38%) | 1196679 (2.49%) | 46489365 (96.87%) |
| HAE-1 | 1860219 (3.83%) | 45517450 (93.68%) | 1250219 (2.62%) | 46727989 (96.17%) |
| HAE-2 | 1499911 (3.10%) | 45638600 (94.35%) | 1232429 (2.55%) | 46871029 (96.90%) |
| HAE-3 | 1809750 (3.86%) | 43908952 (93.69%) | 1146938 (2.45%) | 45055890 (96.14%) |

**Table S5.** The intersection of potential targets for HAE flavonoids and lactation-related targets.

| Number | Gene Symbol | Description | Uniprot ID | Length |
|---|---|---|---|---|
| 1 | FLI1 | Friend leukemia integration 1 transcription factor | Q01543 | 452 |
| 2 | ACHE | Acetylcholinesterase | P22303 | 614 |
| 3 | STAT5A | Signal transducer and activator of transcription 5A | P42229 | 794 |
| 4 | NQO1 | NAD(P)H dehydrogenase | P15559 | 274 |
| 5 | GSR | Glutathione reductase | P00390 | 522 |
| 6 | ETS1 | Protein C-ets-1 | P14921 | 441 |
| 7 | LIF | Leukemia inhibitory factor | P15018 | 202 |
| 8 | ALK | ALK tyrosine kinase receptor | Q9UM73 | 1620 |
| 9 | CCND1 | G1/S-specific cyclin-D1 | P24385 | 295 |
| 10 | ALB | Albumin | P02768 | 609 |
| 11 | GALK1 | Galactokinase | P51570 | 392 |
| 12 | DLD | Dihydrolipoyl dehydrogenase | P09622 | 509 |
| 13 | PIK3CA | Phosphatidylinositol 4,5-bisphosphate 3-kinase catalytic subunit alpha | P42336 | 1068 |
| 14 | BCL2 | Apoptosis regulator Bcl-2 | P10415 | 239 |
| 15 | ESR1 | Estrogen receptor | P03372 | 595 |
| 16 | ERBB4 | Receptor tyrosine-protein kinase erbB-4 | Q15303 | 1308 |
| 17 | TP53 | Cellular tumor antigen p53 | P04637 | 393 |
| 18 | MKI67 | Proliferation marker protein Ki-67 | P46013 | 3256 |
| 19 | ALAD | Delta-aminolevulinic acid dehydratase | P13716 | 330 |
| 20 | CHAT | Choline O-acetyltransferase | P28329 | 748 |
| 21 | MECR | Enoyl-[acyl-carrier-protein] reductase | Q9BV79 | 373 |
| 22 | RBP4 | Retinol-binding protein 4 | P02753 | 201 |
| 23 | FABP2 | Fatty acid-binding protein | P12104 | 132 |
| 24 | VWF | von Willebrand factor | P04275 | 2813 |
| 25 | AR | Androgen receptor | P10275 | 920 |
| 26 | GPI | Glucose-6-phosphate isomerase | P06744 | 558 |
| 27 | ME1 | NADP-dependent malic enzyme | P48163 | 572 |
| 28 | VDR | Vitamin D3 receptor | P11473 | 427 |
| 29 | UQCRC1 | Cytochrome b-c1 complex subunit 1 | P31930 | 480 |
| 30 | CASK | Peripheral plasma membrane protein CASK | O14936 | 926 |
| 31 | IGF1R | Insulin-like growth factor 1 receptor | P08069 | 1367 |
| 32 | GOT2 | Aspartate aminotransferase | P00505 | 430 |
| 33 | HIBCH | 3-hydroxyisobutyryl-CoA hydrolase | Q6NVY1 | 386 |
| 34 | ASS1 | Argininosuccinate synthase | P00966 | 412 |
| 35 | HSPA8 | Heat shock cognate 71 kDa protein | P11142 | 646 |
| 36 | ELF5 | ETS-related transcription factor Elf-5 | Q9UKW6 | 265 |
| 37 | GOT1 | Aspartate aminotransferase | P17174 | 413 |
| 38 | LDHA | L-lactate dehydrogenase A chain | P00338 | 332 |
| 39 | PYGL | Glycogen phosphorylase | P06737 | 847 |

**Table S5** (*continued*)

| Number | Gene Symbol | Description | Uniprot ID | Length |
|---|---|---|---|---|
| 40 | NGLY1 | Peptide-N(4)-(N-acetyl-beta-glucosaminyl)asparagine amidase | Q96IV0 | 654 |
| 41 | HAO1 | 2-Hydroxyacid oxidase 1 | Q9UJM8 | 370 |
| 42 | FBP1 | Fructose-1,6-bisphosphatase 1 | P09467 | 338 |
| 43 | UEVLD | Ubiquitin-conjugating enzyme E2 variant 3 | Q8IX04 | 471 |
| 44 | LDHB | L-lactate dehydrogenase B chain | P07195 | 334 |
| 45 | RPS27A | Ubiquitin-ribosomal protein eS31 fusion protein | P62979 | 156 |
| 46 | ACP3 | Prostatic acid phosphatase | P15309 | 386 |
| 47 | VEGFA | Vascular endothelial growth factor A | P15692 | 395 |
| 48 | MMP1 | Interstitial collagenase | P03956 | 469 |
| 49 | INS | Insulin | P01308 | 110 |
| 50 | GPX3 | Glutathione peroxidase 3 | P22352 | 226 |
| 51 | MMP14 | Matrix metalloproteinase-14 | P50281 | 582 |
| 52 | MIP | Lens fiber major intrinsic protein | P30301 | 263 |
| 53 | DAG1 | Dystroglycan 1 | Q14118 | 895 |
| 54 | TPI1 | Triosephosphate isomerase | P60174 | 249 |
| 55 | GLUL | Glutamine synthetase | P15104 | 373 |
| 56 | AGT | Angiotensinogen | P01019 | 476 |
| 57 | FN1 | Fibronectin | P02751 | 2477 |
| 58 | CDKN2A | Tumor suppressor ARF (Alternative reading frame) | Q8N726 | 132 |
| 59 | B2M | Beta-2-microglobulin | P61769 | 119 |
| 60 | PNPT1 | Polyribonucleotide nucleotidyltransferase 1 | Q8TCS8 | 783 |
| 61 | MDH2 | Malate dehydrogenase | P40926 | 338 |
| 62 | TKT | Transketolase | P29401 | 623 |
| 63 | ANXA5 | Annexin A5 | P08758 | 320 |
| 64 | TTR | Transthyretin | P02766 | 147 |
| 65 | SCO2 | Protein SCO2 homolog | O43819 | 266 |

**Table S6.** KEGG enrichment analysis of the intersection of potential targets for HAE flavonoids and lactation-related targets.

| Term | Pathway | Z-score | p-value | Count | Symbol |
|---|---|---|---|---|---|
| hsa01200 | Carbon metabolism | 21.71 | 2.83E-07 | 11 | DLD, FBP1, GOT1, GOT2, GPI, MDH2, ME1, TKT, TPI1, HIBCH, HAO1 |
| hsa05215 | Prostate cancer | 14.93 | 2.04E-04 | 7 | AR, CCND1, BCL2, IGF1R, INS, PIK3CA, TP53 |
| hsa04933 | AGE-RAGE signaling pathway in diabetic complications | 14.69 | 2.13E-04 | 7 | AGT, CCND1, BCL2, FN1, PIK3CA, STAT5A, VEGFA |
| hsa04066 | HIF-1 signaling pathway | 14.03 | 3.17E-04 | 7 | BCL2, IGF1R, INS, LDHA, LDHB, PIK3CA, VEGFA |
| hsa04151 | PI3K-Akt signaling pathway | 10.59 | 3.52E-04 | 10 | CCND1, BCL2, ERBB4, FN1, IGF1R, INS, PIK3CA, TP53, VEGFA, VWF |
| hsa00010 | Glycolysis / Gluconeogenesis | 15.48 | 4.07E-04 | 6 | DLD, FBP1, GPI, LDHA, LDHB, TPI1 |
| hsa04917 | Prolactin signaling pathway | 15.13 | 4.45E-04 | 6 | CCND1, ELF5, ESR1, INS, PIK3CA, STAT5A |
| hsa01230 | Biosynthesis of amino acids | 14.59 | 5.18E-04 | 6 | ASS1, GLUL, GOT1, GOT2, TKT, TPI1 |
| hsa05207 | Chemical carcinogenesis - receptor activation | 11.24 | 5.58E-04 | 8 | AR, CCND1, BCL2, ESR1, PIK3CA, STAT5A, VDR, VEGFA |
| hsa00620 | Pyruvate metabolism | 15.46 | 9.65E-04 | 5 | DLD, LDHA, LDHB, MDH2, ME1 |
| hsa00270 | Cysteine and methionine metabolism | 14.66 | 1.13E-03 | 5 | GOT1, GOT2, LDHA, LDHB, MDH2 |
| hsa00220 | Arginine biosynthesis | 18.22 | 1.27E-03 | 4 | ASS1, GLUL, GOT1, GOT2 |
| hsa04510 | Focal adhesion | 9.99 | 1.80E-03 | 7 | CCND1, BCL2, FN1, IGF1R, PIK3CA, VEGFA, VWF |
| hsa05230 | Central carbon metabolism in cancer | 12.54 | 2.06E-03 | 5 | LDHA, LDHB, PIK3CA, TP53, SCO2 |
| hsa00630 | Glyoxylate and dicarboxylate metabolism | 15.54 | 2.14E-03 | 4 | DLD, GLUL, MDH2, HAO1 |
| hsa05418 | Fluid shear stress and atherosclerosis | 10.48 | 2.19E-03 | 6 | ASS1, BCL2, NQO1, PIK3CA, TP53, VEGFA |
| hsa00640 | Propanoate metabolism | 15.03 | 2.25E-03 | 4 | DLD, LDHA, LDHB, HIBCH |
| hsa00250 | Alanine, aspartate and glutamate metabolism | 13.94 | 2.74E-03 | 4 | ASS1, GLUL, GOT1, GOT2 |
| hsa05225 | Hepatocellular carcinoma | 9.43 | 3.05E-03 | 6 | CCND1, CDKN2A, NQO1, IGF1R, PIK3CA, TP53 |
| hsa04152 | AMPK signaling pathway | 9.33 | 4.94E-03 | 5 | CCND1, FBP1, IGF1R, INS, PIK3CA |
| hsa04213 | Longevity regulating pathway - multiple species | 10.72 | 5.57E-03 | 4 | HSPA8, IGF1R, INS, PIK3CA |
| hsa05224 | Breast cancer | 8.37 | 6.72E-03 | 5 | CCND1, ESR1, IGF1R, PIK3CA, TP53 |
| hsa04115 | p53 signaling pathway | 9.66 | 7.31E-03 | 4 | CCND1, BCL2, CDKN2A, TP53 |
| hsa04918 | Thyroid hormone synthesis | 9.59 | 7.45E-03 | 4 | ALB, GPX3, GSR, TTR |

**Table S6** (*continued*)

| Term | Pathway | Z-score | p-value | Count | Symbol |
|---|---|---|---|---|---|
| hsa04218 | Cellular senescence | 8.09 | 7.55E-03 | 5 | CCND1, CDKN2A, ETS1, PIK3CA, TP53 |
| hsa04630 | JAK-STAT signaling pathway | 7.81 | 8.77E-03 | 5 | CCND1, BCL2, LIF, PIK3CA, STAT5A |
| hsa00030 | Pentose phosphate pathway | 11.39 | 1.02E-02 | 3 | FBP1, GPI, TKT |
| hsa04211 | Longevity regulating pathway | 8.74 | 1.03E-02 | 4 | IGF1R, INS, PIK3CA, TP53 |
| hsa04010 | MAPK signaling pathway | 6.70 | 1.07E-02 | 6 | ERBB4, HSPA8, IGF1R, INS, TP53, VEGFA |
| hsa01210 | 2-Oxocarboxylic acid metabolism | 11.03 | 1.10E-02 | 3 | DLD, GOT1, GOT2 |

**Table S7.** KEGG enrichment analysis of the potential core targets.

| Term | Pathway | Z-score | p-value | Count | Symbol |
|---|---|---|---|---|---|
| hsa00270 | Carbon metabolism | 26.39 | 1.59E-07 | 9 | DLD, FBP1, GOT1, GOT2, GPI, MDH2, ME1, TKT, TPI1 |
| hsa05224 | Pathways in cancer | 15.97 | 3.44E-07 | 12 | ALK, AR, CCND1, BCL2, CDKN2A, ESR1, FN1, IGF1R, MMP1, PIK3CA, STAT5A, TP53 |
| hsa04115 | Prostate cancer | 22.32 | 1.24E-06 | 7 | AR, CCND1, BCL2, IGF1R, INS, PIK3CA, TP53 |
| hsa04510 | Biosynthesis of amino acids | 21.78 | 4.14E-06 | 6 | ASS1, GLUL, GOT1, GOT2, TKT, TPI1 |
| hsa04211 | Arginine biosynthesis | 26.97 | 1.61E-05 | 4 | ASS1, GLUL, GOT1, GOT2 |
| hsa05417 | Glycolysis/Gluconeogenesis | 19.18 | 1.66E-05 | 5 | DLD, FBP1, GPI, LDHA, TPI1 |
| hsa00630 | Prolactin signaling pathway | 18.76 | 1.76E-05 | 5 | CCND1, ESR1, INS, PIK3CA, STAT5A |
| hsa00030 | AGE-RAGE signaling pathway in diabetic complications | 15.61 | 4.84E-05 | 5 | CCND1, BCL2, FN1, PIK3CA, STAT5A |
| hsa01210 | PI3K-Akt signaling pathway | 11.22 | 4.91E-05 | 7 | CCND1, BCL2, FN1, IGF1R, INS, PIK3CA, TP53 |
| hsa04919 | Alanine, aspartate and glutamate metabolism | 20.73 | 4.92E-05 | 4 | ASS1, GLUL, GOT1, GOT2 |
| hsa04068 | Chemical carcinogenesis - receptor activation | 12.69 | 5.04E-05 | 6 | AR, CCND1, BCL2, ESR1, PIK3CA, STAT5A |
| hsa04910 | HIF-1 signaling pathway | 14.92 | 5.53E-05 | 5 | BCL2, IGF1R, INS, LDHA, PIK3CA |
| hsa04915 | AMPK signaling pathway | 14.13 | 7.78E-05 | 5 | CCND1, FBP1, IGF1R, INS, PIK3CA |
| hsa04218 | Pyruvate metabolism | 18.35 | 8.01E-05 | 4 | DLD, LDHA, MDH2, ME1 |
| hsa04630 | Cysteine and methionine metabolism | 17.42 | 1.13E-04 | 4 | GOT1, GOT2, LDHA, MDH2 |
| hsa05213 | Breast cancer | 12.76 | 1.45E-04 | 5 | CCND1, ESR1, IGF1R, PIK3CA, TP53 |
| hsa05221 | p53 signaling pathway | 14.53 | 2.87E-04 | 4 | CCND1, BCL2, CDKN2A, TP53 |
| hsa05230 | Focal adhesion | 10.74 | 4.79E-04 | 5 | CCND1, BCL2, FN1, IGF1R, PIK3CA |
| hsa05169 | Longevity regulating pathway | 13.20 | 5.03E-04 | 4 | IGF1R, INS, PIK3CA, TP53 |
| hsa01521 | Lipid and atherosclerosis | 10.42 | 5.32E-04 | 5 | BCL2, HSPA8, MMP1, PIK3CA, TP53 |
| hsa05163 | Glyoxylate and dicarboxylate metabolism | 17.25 | 7.80E-04 | 3 | DLD, GLUL, MDH2 |
| hsa04914 | Pentose phosphate pathway | 16.96 | 8.15E-04 | 3 | FBP1, GPI, TKT |

**Table S7** (*continued*)

| Term | Pathway | Z-score | p-value | Count | Symbol |
|---|---|---|---|---|---|
| hsa04922 | 2-Oxocarboxylic acid metabolism | 16.43 | 9.26E-04 | 3 | DLD, GOT1, GOT2 |
| hsa04931 | Thyroid hormone signaling pathway | 11.24 | 1.08E-03 | 4 | CCND1, ESR1, PIK3CA, TP53 |
| hsa04010 | FoxO signaling pathway | 10.77 | 1.31E-03 | 4 | CCND1, IGF1R, INS, PIK3CA |
| hsa04722 | Insulin signaling pathway | 10.52 | 1.45E-03 | 4 | FBP1, INS, PIK3CA, PYGL |
| hsa04071 | Estrogen signaling pathway | 10.52 | 1.45E-03 | 4 | BCL2, ESR1, HSPA8, PIK3CA |
| hsa04114 | Cellular senescence | 9.81 | 2.21E-03 | 4 | CCND1, CDKN2A, PIK3CA, TP53 |
| hsa05165 | JAK-STAT signaling pathway | 9.49 | 2.53E-03 | 4 | CCND1, BCL2, PIK3CA, STAT5A |
| hsa04210 | Endometrial cancer | 12.30 | 2.85E-03 | 3 | CCND1, PIK3CA, TP53 |

**Table S8.** Topological parameters of the potential core flavonoids in the ITP network.

| Number | Compound name | Degree | Betweenness Centrality | Closeness Centrality |
|---|---|---|---|---|
| 1 | Hexamethylquercetagetin | 16 | 0.1638 | 0.4196 |
| 2 | Cichoriin | 15 | 0.2083 | 0.3048 |
| 3 | Paeonoside | 11 | 0.1191 | 0.3652 |
| 4 | Jaceidin | 11 | 0.0667 | 0.3313 |
| 5 | Catechin | 11 | 0.0756 | 0.3120 |
| 6 | Kaempferol | 11 | 0.0734 | 0.3397 |
| 7 | Tangeritin | 9 | 0.0600 | 0.3397 |
| 8 | Limocitrin | 7 | 0.0519 | 0.3441 |
| 9 | Eriodictyol | 7 | 0.0264 | 0.3375 |
| 10 | Hesperetin | 7 | 0.0252 | 0.3375 |
| 11 | Tricetin | 6 | 0.0365 | 0.3375 |
| 12 | Sakuranetin | 5 | 0.0136 | 0.3120 |
| 13 | Quercetin | 5 | 0.0105 | 0.2794 |
| 14 | Naringenin | 5 | 0.0278 | 0.3120 |
| 15 | Gnaphaliin | 4 | 0.0182 | 0.3272 |
| 16 | Isorhamnetin | 4 | 0.0056 | 0.3292 |
| 17 | 3',8-Dihydroxy-4',5',7-trimethoxyflavone | 3 | 0.0190 | 0.2932 |
| 18 | Diosmetin | 3 | 0.0015 | 0.3084 |
| 19 | 3,3',4',5,6,8-Hexamethoxyflavone | 3 | 0.0077 | 0.3156 |
| 20 | Flavone | 3 | 0.0025 | 0.2629 |
| 21 | 4',5,7-Trimethoxyflavone | 2 | 0.0002 | 0.3066 |
| 22 | Sinensetin | 2 | 0.0002 | 0.3066 |
| 23 | 3-Methoxynobiletin | 2 | 0.0002 | 0.3066 |

**Table S9.** Topological parameters of the potential core targets in the ITP network.

| Number | Symbol | Degree | Betweenness Centrality | Closeness Centrality |
|---|---|---|---|---|
| 1 | STAT5A | 22 | 2.41E-01 | 4.30E-01 |
| 2 | PIK3CA | 14 | 3.88E-02 | 3.38E-01 |
| 3 | IGF1R | 12 | 6.06E-02 | 3.58E-01 |
| 4 | TP53 | 11 | 7.35E-02 | 3.51E-01 |
| 5 | CCND1 | 11 | 1.87E-02 | 3.23E-01 |
| 6 | BCL2 | 10 | 1.88E-02 | 3.25E-01 |
| 7 | INS | 8 | 1.26E-01 | 3.49E-01 |
| 8 | AR | 7 | 2.17E-02 | 3.21E-01 |
| 9 | DLD | 7 | 4.26E-02 | 3.35E-01 |
| 10 | GOT1 | 6 | 1.94E-02 | 2.98E-01 |
| 11 | GOT2 | 6 | 2.59E-02 | 3.01E-01 |
| 12 | ESR1 | 6 | 7.18E-03 | 3.14E-01 |
| 13 | FN1 | 5 | 7.87E-03 | 3.18E-01 |
| 14 | LDHA | 5 | 2.11E-02 | 3.19E-01 |
| 15 | ASS1 | 5 | 1.81E-02 | 2.93E-01 |
| 16 | GLUL | 4 | 1.26E-02 | 2.96E-01 |
| 17 | TPI1 | 4 | 1.45E-02 | 3.14E-01 |
| 18 | FBP1 | 4 | 2.78E-02 | 3.03E-01 |
| 19 | MDH2 | 4 | 2.60E-02 | 2.76E-01 |
| 20 | GPI | 4 | 5.60E-03 | 2.68E-01 |
| 21 | CDKN2A | 3 | 4.99E-03 | 3.12E-01 |
| 22 | TKT | 3 | 2.55E-02 | 2.81E-01 |
| 23 | MMP1 | 3 | 4.00E-02 | 3.18E-01 |
| 24 | ME1 | 3 | 7.35E-03 | 2.98E-01 |
| 25 | ALB | 3 | 9.68E-03 | 3.21E-01 |
| 26 | ALK | 3 | 2.88E-03 | 3.12E-01 |
| 27 | HSPA8 | 2 | 1.70E-03 | 2.87E-01 |
| 28 | ANXA5 | 1 | 0.00E+00 | 2.34E-01 |
| 29 | PYGL | 1 | 0.00E+00 | 2.34E-01 |
| 30 | GSR | 1 | 0.00E+00 | 2.38E-01 |

**Table S10.** Quality control of all sample summary in the CK group vs the HQ group.

| Sample | RawReads | RawBases(G) | CleanReads | CleanBases(G) | Q30(%) | GC(%) |
|---|---|---|---|---|---|---|
| CK-1 | 48261916 | 7.24 | 48002428 | 7.17 | 92.81% | 51.56% |
| CK-2 | 48438562 | 7.27 | 48184804 | 7.2 | 93.00% | 51.68% |
| CK-3 | 48515642 | 7.28 | 48223154 | 7.22 | 92.35% | 52.03% |
| HQ-1 | 54100710 | 8.12 | 53796286 | 8.05 | 90.82% | 52.00% |
| HQ-2 | 59267884 | 8.89 | 58997690 | 8.83 | 92.81% | 52.04% |
| HQ-3 | 49278096 | 7.39 | 49021006 | 7.34 | 92.78% | 52.67% |

**Table S11.** Comparison results of sequencing reads and reference genome in the CK group vs the HQ group.

| Sample | Unmapped(%) | Unique Mapped(%) | Multiple Mapped(%) | Total Mapped(%) |
|---|---|---|---|---|
| CK-1 | 1341940 (2.81%) | 45152859 (94.57%) | 1250219 (2.62%) | 46403078 (97.19%) |
| CK-2 | 1397362 (2.91%) | 45363928 (94.57%) | 1209788 (2.52%) | 46573716 (97.09%) |
| CK-3 | 1501961 (3.13%) | 45292686 (94.38%) | 1196679 (2.49%) | 46489365 (96.87%) |
| HQ-1 | 1984621 (3.71%) | 50152010 (93.76%) | 1355153 (2.53%) | 51507163 (96.29%) |
| HQ-2 | 1603679 (2.73%) | 55548257 (94.72%) | 1491212 (2.54%) | 57039469 (97.27%) |
| HQ-3 | 1380234 (2.83%) | 46149283 (94.64%) | 1233861 (2.53%) | 47383144 (97.17%) |

(A)

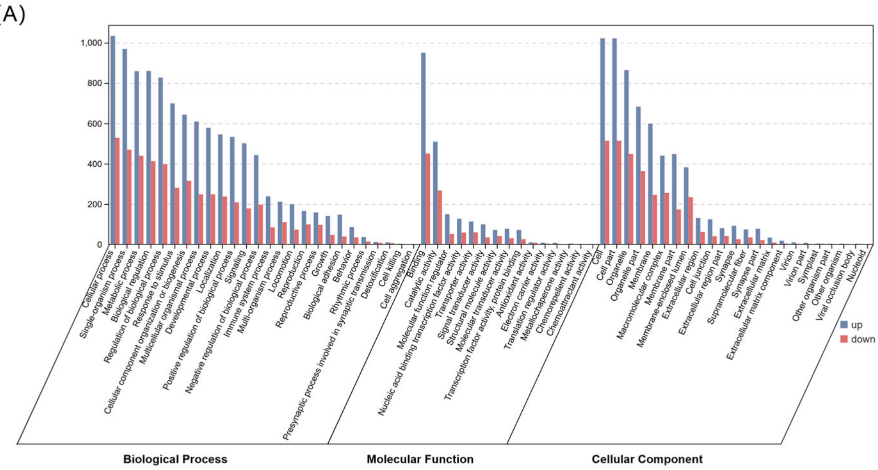

0
200
400
600
800
1,000
Cellular process
Single-organism process
Metabolic process
Biological regulation
Regulation of biological process
Response to stimulus
Cellular component organization or biogenesis
Multicellular organismal process
Developmental process
Localization
Positive regulation of biological process
Signaling
Negative regulation of biological process
Immune system process
Multi-organism process
Locomotion
Reproduction
Reproductive process
Growth
Biological adhesion
Behavior
Rhythmic process
Presynaptic process involved in synaptic transmission
Detoxification
Cell killing
Cell aggregation
Binding
Catalytic activity
Molecular function regulator
Nucleic acid binding transcription factor activity
Transporter activity
Signal transducer activity
Structural molecule activity
Molecular transducer activity
Transcription factor activity, protein binding
Antioxidant activity
Electron carrier activity
Translation regulator activity
Metallochaperone activity
Chemorepellent activity
Chemoattractant activity
Cell
Cell part
Organelle
Organelle part
Membrane
Macromolecular complex
Membrane part
Membrane-enclosed lumen
Extracellular region
Cell junction
Extracellular region part
Synapse
Supramolecular fiber
Synapse part
Extracellular matrix
Extracellular matrix component
Virion
Virion part
Symplast
Other organism part
Other organism
Viral occlusion body
Nucleoid
up
down
Biological Process
Molecular Function
Cellular Component


(B)

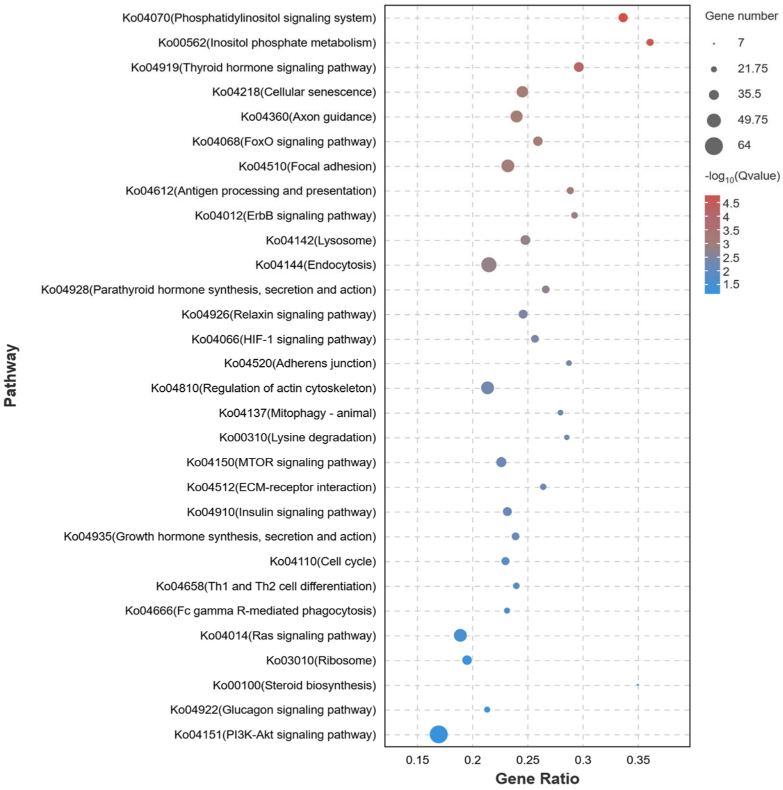

Pathway
Ko04070(Phosphatidylinositol signaling system)
Ko00562(Inositol phosphate metabolism)
Ko04919(Thyroid hormone signaling pathway)
Ko04218(Cellular senescence)
Ko04360(Axon guidance)
Ko04068(FoxO signaling pathway)
Ko04510(Focal adhesion)
Ko04612(Antigen processing and presentation)
Ko04012(ErbB signaling pathway)
Ko04142(Lysosome)
Ko04144(Endocytosis)
Ko04928(Parathyroid hormone synthesis, secretion and action)
Ko04926(Relaxin signaling pathway)
Ko04066(HIF-1 signaling pathway)
Ko04520(Adherens junction)
Ko04810(Regulation of actin cytoskeleton)
Ko04137(Mitophagy - animal)
Ko00310(Lysine degradation)
Ko04150(MTOR signaling pathway)
Ko04512(ECM-receptor interaction)
Ko04910(Insulin signaling pathway)
Ko04935(Growth hormone synthesis, secretion and action)
Ko04110(Cell cycle)
Ko04658(Th1 and Th2 cell differentiation)
Ko04666(Fc gamma R-mediated phagocytosis)
Ko04014(Ras signaling pathway)
Ko03010(Ribosome)
Ko00100(Steroid biosynthesis)
Ko04922(Glucagon signaling pathway)
Ko04151(PI3K-Akt signaling pathway)
0.15
0.2
0.25
0.3
0.35
Gene Ratio
Gene number
7
21.75
35.5
49.75
64
-log10(Qvalue)
4.5
4
3.5
3
2.5
2
1.5


**Figure S1**

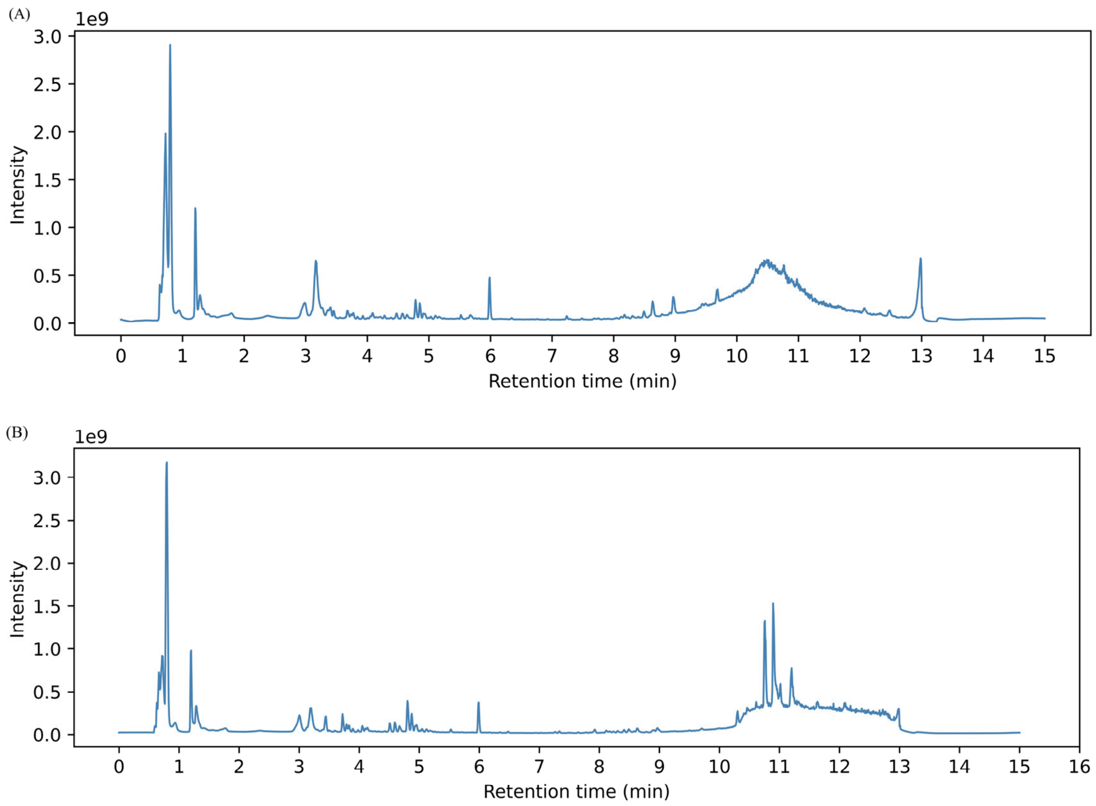

(A)
1e9
3.0
2.5
2.0
1.5
1.0
0.5
0.0
Intensity
0 1 2 3 4 5 6 7 8 9 10 11 12 13 14 15
Retention time (min)
(B)
1e9
3.0
2.5
2.0
1.5
1.0
0.5
0.0
Intensity
0 1 2 3 4 5 6 7 8 9 10 11 12 13 14 15 16
Retention time (min)


**Figure S2**

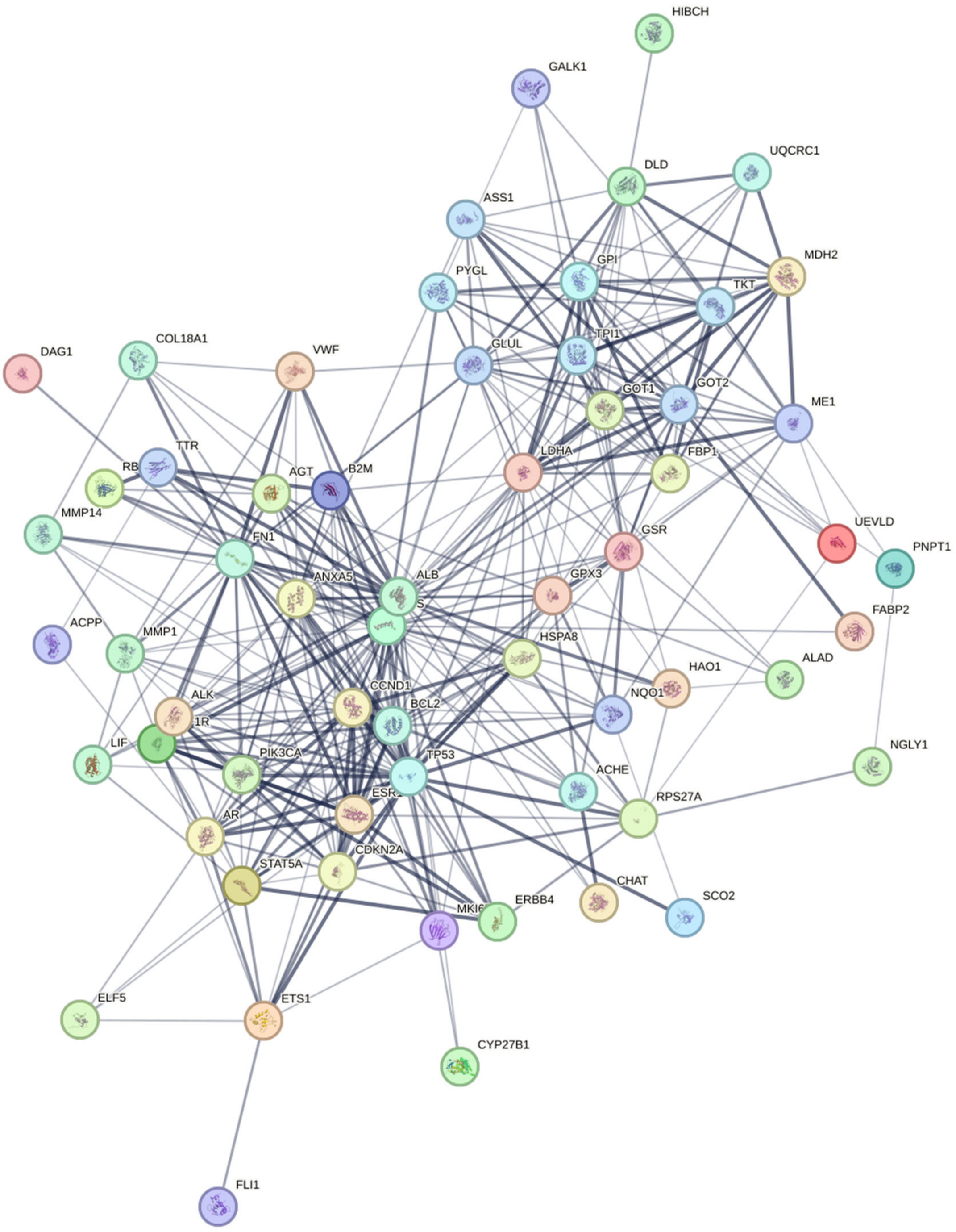
HIBCH
GALK1
UQCRC1
DLD
ASS1
MDH2
PYGL
GPI
TKT
DAG1
COL18A1
VWF
GLUL
TPI1
GOT1
GOT2
ME1
TTR
RB
AGT
B2M
LDHA
FBP1
MMP14
FN1
GSR
UEVLD
PNPT1
ANXA5
ALB
GPX3
FABP2
ACPP
MMP1
HSPA8
ALAD
HAO1
ALK
CCND1
NQO1
BCL2
LIF
PIK3CA
TP53
NGLY1
ACHE
RPS27A
AR
STAT5A
CDKN2A
CHAT
SCO2
ERBB4
ELF5
ETS1
CYP27B1
FLI1

**Figure S3**

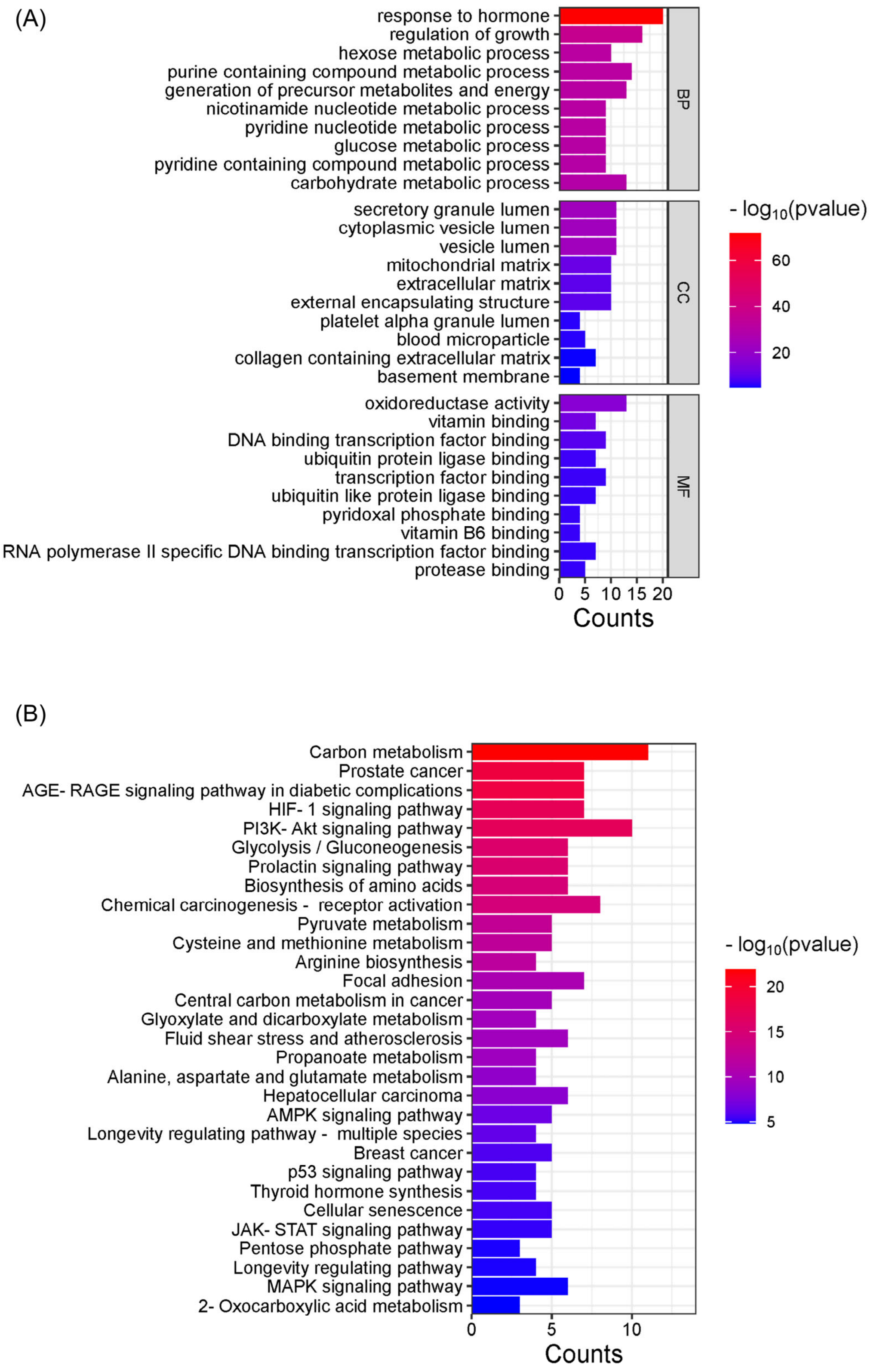

(A)
response to hormone
regulation of growth
hexose metabolic process
purine containing compound metabolic process
generation of precursor metabolites and energy
nicotinamide nucleotide metabolic process
pyridine nucleotide metabolic process
glucose metabolic process
pyridine containing compound metabolic process
carbohydrate metabolic process
BP
secretory granule lumen
cytoplasmic vesicle lumen
vesicle lumen
mitochondrial matrix
extracellular matrix
external encapsulating structure
platelet alpha granule lumen
blood microparticle
collagen containing extracellular matrix
basement membrane
CC
oxidoreductase activity
vitamin binding
DNA binding transcription factor binding
ubiquitin protein ligase binding
transcription factor binding
ubiquitin like protein ligase binding
pyridoxal phosphate binding
vitamin B6 binding
RNA polymerase II specific DNA binding transcription factor binding
protease binding
MF
0 5 10 15 20
Counts
- log10(pvalue)
60
40
20
(B)
Carbon metabolism
Prostate cancer
AGE- RAGE signaling pathway in diabetic complications
HIF- 1 signaling pathway
PI3K- Akt signaling pathway
Glycolysis / Gluconeogenesis
Prolactin signaling pathway
Biosynthesis of amino acids
Chemical carcinogenesis - receptor activation
Pyruvate metabolism
Cysteine and methionine metabolism
Arginine biosynthesis
Focal adhesion
Central carbon metabolism in cancer
Glyoxylate and dicarboxylate metabolism
Fluid shear stress and atherosclerosis
Propanoate metabolism
Alanine, aspartate and glutamate metabolism
Hepatocellular carcinoma
AMPK signaling pathway
Longevity regulating pathway - multiple species
Breast cancer
p53 signaling pathway
Thyroid hormone synthesis
Cellular senescence
JAK- STAT signaling pathway
Pentose phosphate pathway
Longevity regulating pathway
MAPK signaling pathway
2- Oxocarboxylic acid metabolism
0 5 10
Counts
- log10(pvalue)
20
15
10
5


**Figure S4**

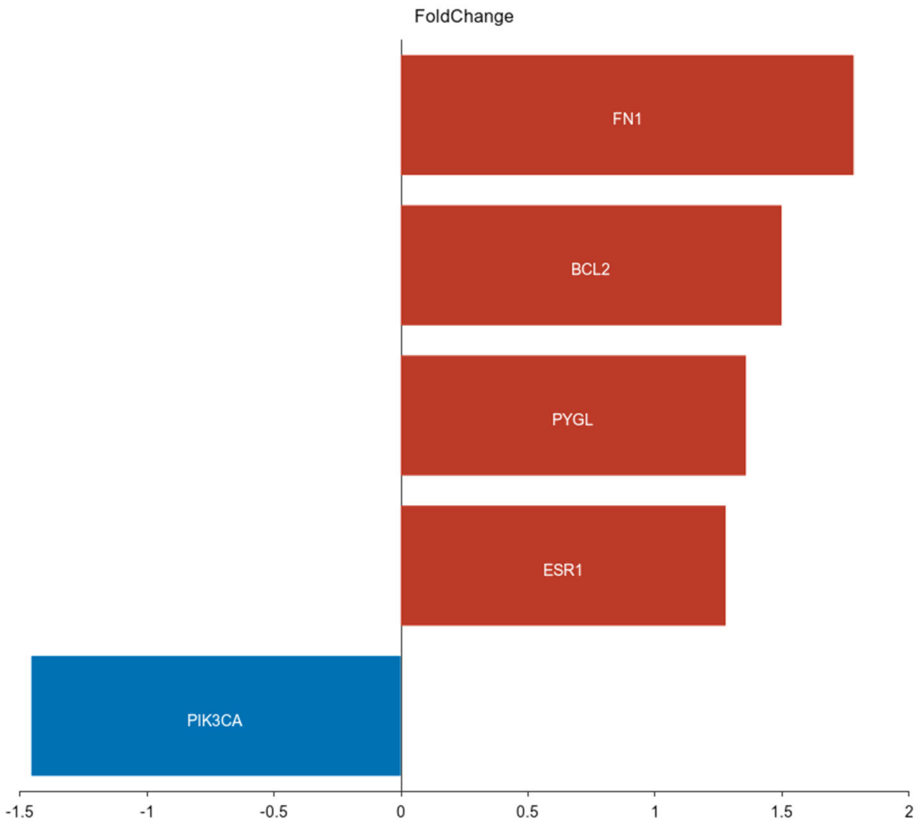


**Figure S5**

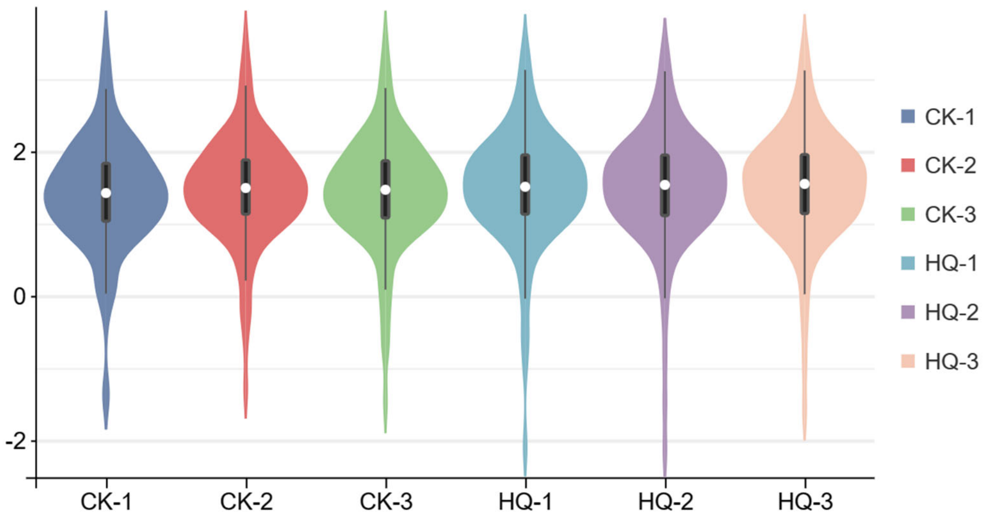


**Figure S6**
